# Supplementary material for: Smoke-free prisons in England: indoor air quality before and after implementation of a comprehensive smoke-free policy
Source: BMJ Open. 2019 Jun 14;9(6):e025782. doi: 10.1136/bmjopen-2018-025782 (PMC6575713; doi:10.1136/bmjopen-2018-025782)
Supplement: Author's manuscript [file bmjopen-2018-025782.draft_revisions.pdf]

BMJ Open

BMJ Open is committed to open peer review. As part of this commitment we make the peer review history of every article we publish publicly available.

When an article is published we post the peer reviewers' comments and the authors' responses online. We also post the versions of the paper that were used during peer review. These are the versions that the peer review comments apply to.

The versions of the paper that follow are the versions that were submitted during the peer review process. They are not the versions of record or the final published versions. They should not be cited or distributed as the published version of this manuscript.

BMJ Open is an open access journal and the full, final, typeset and author-corrected version of record of the manuscript is available on our site with no access controls, subscription charges or pay-per-view fees (http://bmjopen.bmj.com).

If you have any questions on BMJ Open's open peer review process please email editorial.bmjopen@bmj.com

BMJ Open

SMOKE-FREE PRISONS IN ENGLAND: SECOND-HAND SMOKE BEFORE AND AFTER IMPLEMENTING A SMOKE-FREE POLICY

Journal:	*BMJ Open*
Manuscript ID	bmjopen-2018-025782
Article Type:	Research
Date Submitted by the Author:	06-Aug-2018

Complete List of Authors:	Jayes, Leah; The University of Nottingham , Medicine<br>Murray, Rachael; The University of Nottingham , Medicine<br>Opazo Breton, Magdalena; University of Nottingham, Epidemiology and Public Health<br>Hill , Christopher; University of Nottingham School of Medicine, Medicine<br>Ratschen, Elena; University of York, Health Sciences<br>Britton, John; The University of Nottingham

Keywords:	PUBLIC HEALTH, EPIDEMIOLOGY, Adult oncology < ONCOLOGY

SCHOLARONE™
Manuscripts

SMOKE-FREE PRISONS IN ENGLAND: SECOND-HAND SMOKE BEFORE AND AFTER IMPLEMENTING A SMOKE-FREE POLICY

Leah R Jayes[1], Rachael L Murray[1], Magdalena Opazo Breton[1], Christopher Hill[1], Elena Ratschen[2], John Britton[1]

[1]UK Centre for Tobacco and Alcohol Studies and Division of Epidemiology and Public Health, University of Nottingham, Clinical Sciences Building, Nottingham City Hospital, Nottingham, NG5 1PB, UK

[2]Department of Health Sciences, Mental Health and Addictions Research Group, Department of Health Sciences, University of York, York, YO10 5DD, UK

Correspondence to: LR Jayes. leah.jayes@nottingham.ac.uk / +44 115 8231367

For peer review only

Word Count: 3151

Number of Figures: 2

Number of Tables: 2

References: 32

Supplementary Figures: 0

ABSTRACT

Objectives

High levels of particulate pollution due to second-hand smoke (SHS) have previously been recorded in English prisons. As part of an evaluation to ascertain whether the new comprehensive smoke-free policy introduced in the first four prisons in England was successfully implemented, this study compares indoor air quality on prison wing landing locations three months before and three months after going smoke-free.

Setting

The first four prisons in England to implement a comprehensive smoke-free policy.

Primary and secondary measures

We compared concentrations of airborne particulate matter <2.5 microns in diameter ($PM_{2.5}$), as a marker for second-hand smoke, on wing landing locations three months before and three months after the smoke-free policy was implemented.

Results

After discarding data from monitors that had been tampered with we were able to analyse paired data from 74 locations across 29 wing landing locations at the four pilot prisons. When comparing samples taken three months before with the matched samples taken three months after policy implementation, there was a statistically significant ($p<0.001$) 66% reduction in mean $PM_{2.5}$ concentrations at each of the four prisons sampled, from 39.08 μg/m³ to 13.42μg/m³ (difference 25.66 μg/m³, 95% CI 24.95 μg/m³ to 26.37 μg/m³).

Conclusion

For peer review only

Prison smoke-free policies achieve significant improvements in indoor air quality. A national smoke-free policy would therefore be an effective means of protecting prisoners and staff from harm due to SHS exposure in the prison environment.

Strengths and limitations of this study

- This is the first study to compare particulate pollution before and after the implementation of a smoke-free policy in the English prisons.
- The smoke-free policy significantly reduced SHS concentrations, providing HMPPS with strong evidence for it success and continued roll out of smoke-free throughout the rest of the English prison estate.
- Air quality monitoring was not carried out blind, it is possible that prisoners and staff may have changed their behaviour during data collection.
- Pre-policy samples were taken during the winter months and post-policy samples were taken during the summer months, greater ventilation post-policy may have contributed to the reduction in particulate matter.
- Particulate levels post-policy suggest that prisoners were still smoking in some areas, further work is required to investigate and reduce this further.

For peer review only

INTRODUCTION

Since it was introduced a decade ago, smoke-free legislation in the United Kingdom (UK) has been successful in protecting the general public and workforce from harm arising from exposure to second-hand smoke (SHS) (1-3). However the legislation included an exemption for Her Majesty's Prison and Probation Service (HMPPS, formally The National Offender Management Service (NOMS)) (4) which allowed prisoners to smoke in their cells (5), and since around 80% of UK prisoners are smokers (6-9), and many of these are highly tobacco dependent (6, 10), prisoners and prison staff remain at risk of high levels of SHS exposure.

In recent years, HMPPS have come under mounting pressure, from both the Prison Officers' Association (POA, the trade union representing prison officers throughout the UK) (11) and from legal challenges by non-smoking prisoners citing poor health due to personal frequent exposure to SHS (12), to implement a smoke-free policy throughout the prison estate in England and Wales. In September 2015, in response to empirical research demonstrating high levels of SHS in English prisons (13, 14), HMPPS announced the pilot implementation of a comprehensive smoke-free policy in four prisons in the South-West of England (15). This policy prohibited all staff members and prisoners from possessing tobacco or smoking paraphernalia (such as lighters and cigarette rolling paper) within the perimeter walls of the four prisons, but also offered prisoners access to smoking cessation behavioural support and pharmacotherapy, and made disposable electronic cigarettes available for purchase through the prison canteen.

As part of an evaluation of the smoke-free policies introduced in four prisons in the South-West of England in 2016 we have compared indoor air quality, measured as concentrations of airborne particulate matter <2.5 microns in diameter ($PM_{2.5}$), on wing landing locations three months before and three months after going smoke-free. By measuring concentrations of $PM_{2.5}$, this study intends to determine whether the new policy is sufficient in reducing concentrations of SHS.

For peer review only

METHODS

Study prisons

Data were collected from the first four English Prison Service establishments selected to go smoke-free, with one prison going smoke-free every two weeks between 11th April and 23rd May 2016. The prisons were all in the South-West of England, and were selected for reasons including their low transfer rate to other regional areas, being all-male establishments, and having a relatively stable population. According to HMPPS annual performance ratings, all four prisons were performing well at the time of data collection and had reported no recent incidents (16). The local prison (HMP 1) held prisoners on remand or serving sentences, while the other three training establishments (HMPS 2, 3, & 4) only held prisoners who had been sentenced. Further information on the four prisons is presented in Table 1.

Before the smoke-free policy was implemented, all four prisons had a non–smoking policy for staff members within the perimeter wall, while prisoners were allowed to smoke only in their cells. However, smoking still occurred regularly in the exercise yards. The only exception was the residential healthcare unit at HMP 1, which was designated 'smoke-free' and in which all indoor smoking was prohibited. This unit was therefore excluded from our study.

Particulate pollution

The concentration of airborne particulate matter <2.5 microns in diameter ($PM_{2.5}$) is a well-established marker of indoor SHS concentrations (17, 18), and previous studies have shown high $PM_{2.5}$ concentrations in environments where smoking has taken place (18, 19). Eleven battery-operated SidePak Personal Aerosol Monitors AM510 (TSI Inc, MN, USA) fitted with a $PM_{2.5}$ impactor and set to a calibration factor of 0.30, as appropriate for tobacco smoke (20, 21) were used to measure $PM_{2.5}$ concentrations at each prison visit. In accordance with manufacturer's instructions, SidePak devices were cleaned, the impactor re-greased, zero-calibrated and the flow rate set at 1.7

For peer review only

l/min before each use. $PM_{2.5}$ measurements were logged at one minute intervals, with each one minute data point being an average of 60 seconds of sample measurements. Depending on staff escort availability, data were collected over three or four consecutive weekdays, and again if possible on the same weekdays, before and after the smoke-free policy was introduced (see Table 1 for study prison characteristics and data collection dates). Two researchers trained in the use of air quality monitors placed the SidePak monitors in static locations on wing landings. Samples collected were compared with current World Health Organisation (WHO) indoor air quality standards, which recommend that $PM_{2.5}$ concentrations alone should not exceed 25 µg/m³ as a 24-hour mean (22).

Data collection

The four prisons were visited by two researchers three months before and after each prison's smoke-free implementation date (see Table 1). The researchers were assigned a prison officer during their data collection to gain access to all the wing landings to place the SidePak air monitors, and to advise on areas that were not currently accessible (typically due to prisoner incidents). Each air quality sample was identified with a unique code, and details recorded of the date and day of data collection; the prison, wing location and position of monitor; time the monitor was switched on and off; whether there was evidence that the monitor had been moved or tampered with; monitor serial number, and visit number (visit 1 = pre-policy implementation, visit 2= post-policy implementation). The sampling duration of each datasets were determined by access to wings locations in order to collect monitors and the machines battery life (around 11 hours). Wing landings were chosen to cover a range of designs and functions. The monitors were usually placed half way down the wing, above head height and away from open outside doors, windows, or cooking equipment. Where possible, monitors were placed in discreet locations to avoid disrupting prisoners' normal behaviour. Officers on each wing were advised where each monitor had been placed and for how long.

All monitors at HMP 2 were placed at one end of the unit next to the wing office, as air quality monitors had been taken by prisoners during earlier sampling. Due to the landing design of several

For peer review only - http://bmjopen.bmj.com/site/about/guidelines.xhtml

wings at HMP 3, air quality monitors had to be placed in a cupboard which inhibited air flow. Pre-implementation sampling logs and unique codes were used post-implementation to guide repeat data collection; where feasible placing SidePak monitors on the same day of the week, wing location, position on landing, start time and duration of sample.

Patient and Public Involvement

There was no patients' or public involvement in this study.

For peer review only

Table 1. Study prison characteristics and data collection dates

Prison	HMP 1	HMP 2	HMP 3	HMP 4
Category and function*	Male<br>Category B<br>Local	Male<br>Category C<br>Training	Male<br>Category C<br>Training	Male<br>Category C<br>Training
Structural design	Built 1850s<br>Victorian radial design	Built early 1800s<br>Singular wings	Built 1974<br>Five two story living blocks and quick build wings	Built 1960s<br>Mix of triangular, T-shaped and quick build wings
Number of wings	7	7	9	9˜
Prisoner roll count (pre-implementation)	505	634	706	518˜
Sampling dates pre-smoke-free	19/01/16 – 23/01/16	08/02/16 – 11/02/16	15/02/16 – 18/02/16	29/02/16 – 02/03/16
Smoke-free implementation date	11/04/16	25/04/16	09/05/16	23/05/16
Sampling dates post-smoke-free	05/07/16 – 08/07/16	18/07/16 – 21/07/16	22/08/16 – 25/08/16	15/08/16 – 17/08/16

*Category B prisons hold prisoners for whom the very highest conditions of security are not necessary but for whom escape must be made very difficult.
*Category C prisons hold prisoners who cannot be trusted in open conditions but who do not have the resources and will to make a determined escape attempt.
*Local prisons serve the courts and receive remand and post-conviction prisoners prior to their allocation to other establishments.
*Training prisons hold sentenced prisoners who tend to be employed in a variety of activities such as prison workshops, gardens and education and in offending behaviour programme.
˜HMP 4 closed two wings (and transferred all prisoners located on these wings) between pre- and post- smoke-free data sampling dates.

For peer review only

Data analysis

Each dataset was downloaded from the monitor using Trackpro 4.6.1 software and imported into STATA 13, alongside their unique code. Datasets were then paired using their unique code and corresponding sample times matched (to the minute) to compare $PM_{2.5}$ concentrations pre- and post-implementation. Data from monitors that appeared to have been moved or tampered with, and those with no paired sample, were discarded. Descriptive statistics for all paired data and paired data by prison ID were generated; including mean, range, median, interquartile range, and the proportion of time the $PM_{2.5}$ concentration exceeded WHO 24- hour mean $PM_{2.5}$ upper limit of 25 $\mu g/m^3$ (22). The percentage change of $PM_{2.5}$ concentrations was determined by comparing the mean and median $PM_{2.5}$ levels overall and in each prison before and after smoke-free. The Wilcoxon signed-rank test was used to assess statistical significance between pre- and post- implementation $PM_{2.5}$ concentrations in each establishment. To illustrate the sampled $PM_{2.5}$ distribution from each prison before and after implementation of smoke-free, boxplots were constructed. Although $PM_{2.5}$ data distributions were skewed, we present arithmetic mean figures throughout since these are used by the WHO to define their upper guidance limits.

Ethics approval

The University of Nottingham, Medical School Ethics Committee (G06062013 CHS EPH) approved this study, it was then subsequently approved by NOMS, National Research Committee (NRC) (Ref: 2013-202) in July 2014. Permission to enter all four prisons for data collection was sought from the Deputy Director of Public Sector Prisons and the Deputy Director of Custody for the South-West area. The Governors at each prison also agreed to the research being undertaken at their establishments.

For peer review only

RESULTS

A total of 200 datasets were collected from 29 wing landings locations throughout the four prisons. One SidePak monitor was destroyed during pre-implementation data collection, and on 12 occasions monitors were tampered with by prisoners (for example, by blocking the air inlet or turning off the monitor). The remaining 187 datasets included 113 collected before and 74 collected after policy implementation; the lower number after implementation arose primarily from restrictions on access to some prison wings. We therefore generated 74 paired sets of data for analysis. On sampling days both pre- and post-policy implementation all wings, apart from the Care and Separation Unit (CSU), were at or near full capacity, with prisoner occupancy per wing ranging from 19 to 180.

Combined data from all four prisons, comparing $PM_{2.5}$ concentrations collected pre- and post-implementation

Mean $PM_{2.5}$ concentrations on wing landing locations before the introduction of smoke-free policy were 39.08 µg/m³, and 13.42µg/m³ after introduction, representing a 66% reduction in mean $PM_{2.5}$ concentrations (mean difference 25.66 µg/m³, 95% Confidence Intervals 24.95 µg/m³ to 26.37 µg/m³); and a 69% reduction in median $PM_{2.5}$ concentrations (from 26µg/m³ to 8µg/m³). The mean $PM_{2.5}$ concentration pre- implementation exceeded the WHO 24- hour mean $PM_{2.5}$ upper limit of 25 µg/m³ (22), and continuously monitored levels were above this limit for more than half of all sampling time (see Table 2).

For peer review only

Table 2. Summary of sampled $PM_{2.5}$ concentrations combined and individually for four prisons pre- and post- smoke-free implementation.

	**Combined prison data**		**HMP 1**		**HMP 2**		**HMP 3**		**HMP 4**	
**Visit∞**	**1**	**2**	**1**	**2**	**1**	**2**	**1**	**2**	**1**	**2**
**Number of paired datasets (total matched sample time hr:min)**	74<br>380:20		20<br>97:57		14<br>70:57		22<br>125:50		18<br>85:36	
**Arithmetic mean (and range) of $PM_{2.5}$ concentrations (μg/m³)**	39.08<br>(0-1359)	13.42<br>(0-3073)	66.41<br>(2-678)	14.00<br>(0-635)	12.85<br>(0-121)	6.44<br>(0-30)	34.57<br>(0-1359)	14.62<br>(2-227)	36.14<br>(1-1058)	16.78<br>(0-3075)
**Arithmetic mean percentage reduction from pre- to post-implementation**	66%		79%		50%		58%		54%	
**Median (and IQR) of $PM_{2.5}$ concentrations (μg/m³)**	26<br>(15-46)	8<br>(4-15)	42<br>(27-76)	8<br>(4-16)	11<br>(6-17)	6<br>(2-9)	27<br>(17-44)	9<br>(5-17)	29<br>(18-44.5)	8<br>(4-18)
**Median percentage reduction from pre- to post-implementation**	69%		81%		45%		67%		72%	
**Percentage of time above 25 μg/m³^**	51%	11%	20%	3%	1%	0%	18%	5%	13%	3%

∞ Visit number, 1 = pre- smoke-free policy implementation, 2 = post- smoke-free policy implementation

IQR = interquartile range

^ WHO 24- hour mean $PM_{2.5}$ upper limit of 25 μg/m³

For peer review only

Individual data from all four prisons, comparing $PM_{2.5}$ concentrations collected pre- and post-implementation

Data for the four prisons sampled (Table 2) demonstrate that all but HMP 2 had mean $PM_{2.5}$ concentrations above the WHO 24- hour mean upper limit pre-policy implementation, and all had mean post-policy concentrations below this limit (22). All four prisons saw a statistically significant reduction in the $PM_{2.5}$ concentration pre-to post- smoke-free policy (median percentage reductions, HMP1 = 81%, HMP 2 = 45%, HMP 3 = 67%, HMP 4 = 72%, all four prisons, $p<0.001$). HMP 1, the local prison, had the highest mean and median $PM_{2.5}$ concentrations pre-policy, and the largest percentage reduction post- policy for these samples. Excluding HMP 2, the other three prisons lowered the time spent over the WHO 24- hour mean $PM_{2.5}$ upper limit from 13-20% to 3-5%. Figure 1 shows box plots of the distribution of $PM_{2.5}$ concentrations measured in each prison before and after the smoke-free policy. An example of the difference in $PM_{2.5}$ concentration profiles on a main residential wing at HMP 3, pre- and post-implementation, is presented in Figure 2.

For peer review only

Figure 1. Box plots of $PM_{2.5}$ distributions in each of the four prisons pre- and post-smoke-free implementation.

[INSERT FIGURE 1]

The horizontal line in each box represents the median value and the top and bottom of the box represent the 25th and 75th percentile, with the lines extending from the top and bottom of the boxes widening to the 5th and 95th percentile of the distribution. For ease of representation, Figure 1 does not show $PM_{2.5}$ concentrations over 500 µg/m³ (this only applies to samples taken from HMPs 1 & 4).

Figure 2. Concentrations of $PM_{2.5}$ sampled on a main residential wing at HMP 3 pre- and post-smoke-free implementation.

[INSERT FIGURE 2]

For peer review only

DISCUSSION

This is the first study to compare particulate pollution before and after the implementation of smoke-free policy in English prisons. The air quality measures, which used concentrations of $PM_{2.5}$ as a proxy for SHS, demonstrate that before the smoke-free policy was introduced $PM_{2.5}$ levels were well in excess of the WHO 24- hour mean $PM_{2.5}$ upper limit (22), with half of all sampling time over this recommended guidance level. After introduction of the smoke-free policy there was a substantial and statistically significant reduction in $PM_{2.5}$ concentrations, to below the WHO upper guidance limit of 25 μg/m³ per 24-hour. However the range of concentrations sampled suggest that prisoners were still smoking on occasions under the smoke-free policy.

Our air quality measurements were not carried out in blind fashion, because researchers were obliged to answer questions from staff members and prisoners who enquired about the monitoring. However, whilst it is possible that prisoners or staff changed their behaviour in response to being monitored, we think that is unlikely to have occurred to any appreciable degree over the course of our measurements. SHS is not the only source of indoor $PM_{2.5}$, which includes particulate matter released from sources such as open fires, toasters and microwaves. However, where toasters and microwaves were present on the wings, every effort was made to place the SidePak monitors as far away from these as possible. Safe locations for the SidePak monitors were limited, but researchers tried to collect data from all wings at each prison. Since security concerns and the design of the wings at HMP 2 and HMP 3 required us to place the Sidepak monitors in cupboards, these measures are likely to have underestimated the true $PM_{2.5}$ concentrations on these wing locations pre- and post- smoke-free policy. Nevertheless, reductions in $PM_{2.5}$ concentrations were still observed after policy implementation in the majority of these samples. Similar issues with placement of SidePak monitors on wing locations were described in work carried out in a New Zealand prison, but that study also reported a significant reduction in $PM_{2.5}$ concentration after going smoke-free [10].

For peer review only

As an inevitable consequence of the smoke-free implementation dates in the four prisons, pre-policy air quality samples were taken during the winter months and post-policy during the summer months. It is possible that greater ventilation through open windows in the summer months may have contributed to the reduction in particulate levels between these two time points. However, to minimise this bias, SidePak monitors were placed towards the centre of the wings and away from any open windows during sampling. Our data collection was also limited by regime changes or episodes of lock-down due to prisoner incidents, which resulted in fewer samples after the policy was introduced. We are unable to say whether this has significantly biased our findings. We recognise that our estimates of the proportion of time spent above the WHO $PM_{2.5}$ upper guidance limit of 25 µg/m³ as a 24-hour mean are not truly representative because the maximum sampling time was determined by the battery life of the SidePak monitors used (around 11 hours) and we were only able to place the monitors onto the wings during daytime hours. Since smoking does not occur during sleep, particulate levels are likely to have been considerably lower during the night. However our data does give a very good estimation, in view of the large amount of data collected pre- and post- policy (over 15 days pre- and post- policy), of SHS pollution during times when non-smokers would be exposed during waking hours.

In an earlier air quality monitoring study (which included two of the pilot smoke-free sites sampled here), we measured $PM_{2.5}$ concentrations on wing landings where prisoners were permitted to smoke in their cells that were slightly higher than those three months prior to the smoke-free implementation in the present study (mean values 43.87 µg/m³ and 39.08 µg/m³ respectively) (13). A possible explanation for this is that the majority of samples taken in the current study were carried out on days leading up to the weekly delivery of tobacco to prisoners from the prison shop (data in this study were collected Monday-Friday, with canteen delivery typically occurring on Fridays) when many prisoners are running out of tobacco, whereas the earlier study included samples taken at the weekend (after tobacco delivery). This earlier study reported that $PM_{2.5}$ concentrations were higher immediately after canteen delivery days (13). It is also possible however that three months before

For peer review only

going smoke-free, prisoners were already starting to reduce their tobacco consumption or had been on a smoking cessation course at the prison in light of the impending policy.

Since 2005 the USA, New Zealand, Canada, and Australia have all implemented smoke-free policies in their correctional facilities. International air quality studies from New Zealand and the US have shown that comprehensive smoke-free polices are effective in substantially reducing SHS concentrations (23-25). All of these studies used markers of SHS, respirable particulate matter (for example, particulate matter less than 2.5 μg/m³ ($PM_{2.5}$)) (24, 25) and airborne nicotine (23) to sample prison locations pre- and post- policy. The percentage reductions in $PM_{2.5}$ concentrations in our study were very similar to those recorded in these other countries (23-25).

Alongside reduced SHS concentrations, the potential health benefits of introducing a comprehensive smoke-free policy have been outlined in a study which examines the 10 years since the US implemented its smoking ban in prisons. This study found that prisons who implemented a smoke-free policy had a 9% reduction in smoking related deaths (particularly cardiovascular and pulmonary deaths), and that bans in place for longer than nine years were associated with a reduction in cancer deaths (26). A study exploring natural deaths in male prisoners over 60 years of age in England and Wales reported diseases of the circulatory system (such as, coronary heart disease and cerebrovascular disease) and respiratory illnesses, all of which are substantially more common among smokers, as the most common cause of death (32). With these findings in mind, the future roll-out of smoke-free across all 121 prisons in England and Wales set out by HMPPS (15) has the potential to have the same positive health impact on the nearly 83,000 prisoners (27) currently held, and the thirty-two thousand staff members employed (28), in the prison estate.

Findings from this study suggest prisoners were still smoking after the introduction of smoke-free, since $PM_{2.5}$ concentrations post- policy ranged from 0-3073 μg/m³, consistent with continued smoking in some areas. Hammond and colleagues measured nicotine concentrations before and after prisons in California, US went smoke-free, and concluded that a smoking ban was effective in

For peer review only

reducing SHS exposure but did not eliminate it (23). An ethnographic case study conducted in ten prisons in the US after implementing a complete smoking ban described the lengths prisoners would go to in order to acquire, exchange and smoke tobacco, and how tobacco had now become a more lucrative commodity to sell due to big demand and higher profit margin than illicit drugs (29). The study concluded that although prisoners smoked less post-policy, the emergent black-market created by banning tobacco had a negative impact on inmates. The emergence of a tobacco black market was also observed in New Zealand and the Northern Territories of Australia after their implementation of smoke-free (30, 31).

However, despite these potential adverse effects of going smoke-free, our findings suggest that smoke-free policies in these prisons has successfully reduced prisoner smoking, and both prisoner and staff exposure to SHS. Further work to reduce still further the occurrence of prisoner smoking is clearly required, and to assess the impact of smoke-free policy on prisoner health. However our data provide strong evidence in support of extending smoke-free policy throughout the English and Welsh prison estate.

For peer review only

Security clearance

Researchers (LJ & CH) were security cleared to Enhanced Level 1, enabling them to visit establishments and work within NOMS. Researchers were given security talks from each establishments prior to data collection.

Authors' contributors

LJ is the guarantor and takes responsibility for the integrity of the work as a whole, from inception to publication; study conception and design, data collection, analysis of data, interpretation of data and drafting the research manuscript. RM, ER and JB contributed to the study conception and design. CH assisted with data collection and MOB supported data preparation in STATA and data analysis. RM and JB aided drafting the manuscript. All authors read and approved the final manuscript.

Acknowledgements

The lead author would like to thank all four prisons for agreeing to participate in this research study, alongside the staff members who escorted the researchers during data collection.

Funding

This study was funded by a UK Centre for Tobacco and Alcohol Studies (UKCTAS) PhD studentship.

Data Sharing Statement

All available data can be obtained by contacting the corresponding author.

Competing Interests

None declared.

For peer review only

LEGEND

Figure 1. Box plots of $PM_{2.5}$ distributions in each of the four prisons pre- and post-smoke-free implementation.

Figure 2. Concentrations of $PM_{2.5}$ sampled on a main residential wing at HMP 3 pre- and post-smoke-free implementation.

References

1. Bauld L. The impact of smokefree legislation in England: Evidence review. Available from: https://www.gov.uk/government/uploads/system/uploads/attachment_data/file/216319/dh_124959.pdf 2011.

2. Department of Health. Smoke-free England - one year on London: UK: Available from: http://www.smokefreeengland.co.uk/files/dhs01_01-one-year-on-report-final.pdf 2008.

3. Local Government Association. A breath of fresh air: Smokefree workplaces 10 years on. London: UK June 2017

4. HM Government. Health Act 2006. Chapter 28. Great Britain: Smoking; 2006.

5. Prison Service Instruction 09/2007. London: Smoke free legislation: prison service application; 2007.

6. Singleton N, Farrell M, Meltzer H. Substance misuse among prisoners in England and Wales. Available from: http://webarchive.nationalarchives.gov.uk/20160105160709/http://ons.gov.uk/ons/rel/psychiatric-morbidity/substance-misuse-among-prisoners/substance-misuse-among-prisoners/index.html 1999.

7. Department of Health and HM Prison Service. Acquitted: Best practice guidance for developing smoking cessation services in prisons. London: 2003.

For peer review only

8. Lester C, Hamilton-Kirkwood L, Jones N. Health indicators in a prison population: asking prisoners. Health Education Journal 2003;62:341-9.

9. Scottish Prison Service. Prisoner Survey 2015. Scotland Scottish Prison Service 2015.

10. Plugge EH, Foster CE, Yudkin PL, Douglas N. Cardiovascular disease risk factors and women prisoners in the UK: the impact of imprisonment. Health Promotion International. 2009;24(4):334-43.

11. Prison Officers Association. Health and Safety Smoke Free http://www.poauk.org.uk/index.php?circulars&newsdetail=20111114-185_health-safety-smoke-free-prison-estate Prison Circular 2011.

12. Black -v- Secretary of State for Justice (Case No: CO/1258/2014): Hearing before the In The High Court Of Justice,(5 March 2015).

13. Jayes LR, Ratschen E, Murray RL, Dymond-White S, Britton J. Second-hand smoke in four English prisons: an air quality monitoring study. BMC Public Health. 2016;16(1):119.

14. Brinckerhoff P. Report on Second-Hand Smoke in Prisons: Final Report. Available at - https://www.gov.uk/government/publications/air-quality-reports 2015

15. Ministry of Justice. Ministry of Justice, Andrew Selous MP and National Offender Management Service. Letter from Prisons Minister Andrew Selous to Robert Neill MP, Chairman of the Justice Select Committee regarding smoking in prisonsSeptember 2015.

16. The National Offender Management Service (NOMS). Prison Annual Performance Ratings 2015/16. Available from: https://www.gov.uk/government/uploads/system/uploads/attachment_data/file/541173/prison-annual-performance-ratings-2015-2016.pdf: 2015/2016

For peer review only

17. Sureda X, Fernandez E, Lopez MJ. Secondhand tobacco smoke exposure in open and semi-open settings: a systematic review. Environ Health Perspect. 2013;121.

18. Hyland A, Travers MJ, Dresler C. A 32-country comparison of tobacco smoke derived particle levels in indoor public places. Tob Control. 2008;17.

19. Semple S, Apsley A, Galea KS, MacCalman L, Friel B, Snelgrove V. Secondhand smoke in cars: assessing children's potential exposure during typical journey conditions. Tobacco Control. 2012.

20. Semple S, Tongeren M, Galea KS. UK smoke-free legislation: changes in PM2.5 concentrations in bars in Scotland, England, and Wales. Ann Occup Hyg. 2010;54.

21. Jiang RT, Acevedo-Bolton V, Cheng KC. Determination of response of real-time SidePak AM510 monitor to secondhand smoke, other common indoor aerosols, and outdoor aerosol. J Environ Monit. 2011;13.

22. WHO air quality guidelines. Geneva, Switzerland: Global Update; 2005.

23. Hammond SK, Emmons KM. Inmate exposure to secondhand smoke in correctional facilities and the impact of smoking restrictions. J Expo Anal Environ Epidemiol. 2004;15(3):205-11.

24. Proescholdbell SK, Foley KL, Johnson J. Indoor air quality in prisons before and after implementation of a smoking ban law. Tob Control. 2008;17.

25. Thornley S, Dirks KN, Edwards R. Indoor air pollution levels were halved as a result of a national tobacco ban in a New Zealand prison. Nicotine Tob Res. 2013;15.

26. Binswanger I, Carson E, Krueger P, Mueller S, Steiner J, Sabol W. Prison tobacco control policies and deaths from smoking in United States prisons: population based retrospective analysis. BMJ. 2014;349.

27. Ministry of Justice. Her Majesty's Prison and Probation Service. Prison population figures: 2018. Availabe from: https://www.gov.uk/government/statistics/prison-population-figures-2018 2018.

For peer review only

28. Ministry of Justice. Her Majesty's Prison and Probation Service workforce quarterly: March 2018. Available from: https://www.gov.uk/government/statistics/her-majestys-prison-and-probation-service-workforce-quarterly-march-2018 2018.

29. Lankenau SE. Smoke 'em if you got 'em: Cigarette black markets in U.S. prisons and jails. The Prison journal. 2001;81(2):142-61. PubMed PMID: PMC2117377.

30. Collinson L, Wilson N, Edwards R, Thomson G, Thornley S. New Zealand's smokefree prison policy appears to be working well: one year on. The New Zealand Medical Journal. 2012;125(1357):164-8.

31. Hefler M, Hopkins R, Thomas D. Successes and unintended consequences of the Northern Territory's smoke-free prisons policy: results from a process evaluation. Public Health Research & Practice. 2016.

32. Fazel S, Benning R. Natural deaths in male prisoners: a 20-year mortality study. European Journal of Public Health. 2006;16(4):441-4.

For peer review only

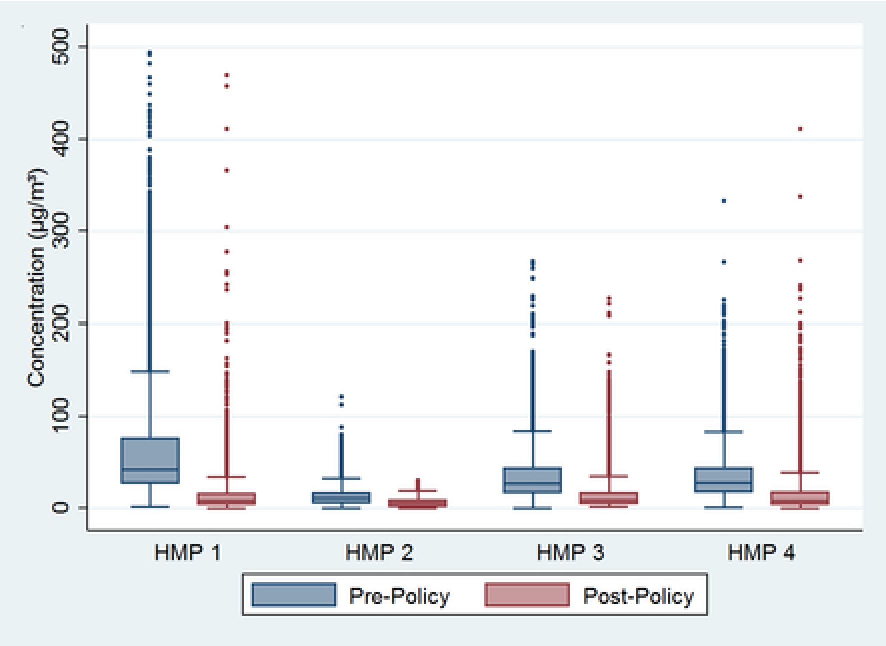

37x27mm (300 x 300 DPI)

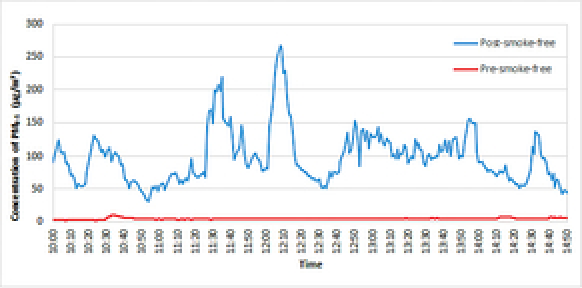

24x12mm (300 x 300 DPI)

BMJ Open

Smoke-free prisons in England: Indoor air quality before and after implementation of a comprehensive smoke-free policy

Journal:	*BMJ Open*
Manuscript ID	bmjopen-2018-025782.R1
Article Type:	Research
Date Submitted by the Author:	14-Jan-2019
Complete List of Authors:	Jayes, Leah; The University of Nottingham , Medicine<br>Murray, Rachael; The University of Nottingham , Medicine<br>Opazo Breton, Magdalena; University of Nottingham, Epidemiology and Public Health<br>Hill , Christopher; University of Nottingham School of Medicine, Medicine<br>Ratschen, Elena; University of York, Health Sciences<br>Britton, John; The University of Nottingham
**Primary Subject Heading**:	Smoking and tobacco
Secondary Subject Heading:	Public health
Keywords:	PUBLIC HEALTH, EPIDEMIOLOGY, Epidemiology < ONCOLOGY

SCHOLARONE™
Manuscripts

Smoke-free prisons in England: Indoor air quality before and after implementation of a comprehensive smoke-free policy

Leah R Jayes[1], Rachael L Murray[1], Magdalena Opazo Breton[1], Christopher Hill[1], Elena Ratschen[2], John Britton[1]

[1]UK Centre for Tobacco and Alcohol Studies and Division of Epidemiology and Public Health, University of Nottingham, Clinical Sciences Building, Nottingham City Hospital, Nottingham, NG5 1PB, UK

[2]Department of Health Sciences, Mental Health and Addictions Research Group, Department of Health Sciences, University of York, York, YO10 5DD, UK

Correspondence to: LR Jayes. leah.jayes@nottingham.ac.uk / +44 115 8231367

Word Count: 4038

Number of Figures: 2

Number of Tables: 2

References: 33

Supplementary Figures: 0

For peer review only

ABSTRACT

Objectives

High levels of particulate pollution due to second-hand smoke (SHS) have previously been recorded in English prisons. As part of an evaluation to ascertain whether a new comprehensive smoke-free policy introduced in the first four prisons in England was successfully implemented, this study compares indoor air quality on prison wing landing locations three months before and three months after going smoke-free.

Design

An indoor air quality monitoring study, comparing SHS levels before and after a comprehensive smoke-free prison policy.

Setting

The first four prisons in England to implement a comprehensive smoke-free policy.

Primary and secondary measures

We compared concentrations of airborne particulate matter <2.5 microns in diameter ($PM_{2.5}$), as a marker for SHS, on wing landing locations three months before and three months after the smoke-free policy was implemented. Static battery operated aerosol monitors were used to sample concentrations of $PM_{2.5}$ on wing landings.

Results

After discarding data from monitors that had been tampered with we were able to analyse paired data across four prisons from 74 locations, across 29 wing landing locations, for an average sampling time of five hours and eight minutes. When comparing samples taken three months before with the paired samples taken three months after policy implementation (paired for prison, day of the week, time of day, wing location and position of monitor), there was a statistically significant ($p<0.001$)

For peer review only

66% reduction in mean $PM_{2.5}$ concentrations at each of the four prisons sampled, from 39 µg/m³ to 13µg/m³ (difference 26 µg/m³, 95% CI 25 µg/m³ to 26 µg/m³).

Conclusion

Prison smoke-free policies achieve significant improvements in indoor air quality. A national smoke-free policy would therefore be an effective means of protecting prisoners and staff from harm due to SHS exposure in the prison environment.

Strengths and limitations of this study

- This is the first study to compare particulate pollution before and after the implementation of a smoke-free policy in English prisons.
- Air quality monitoring was not carried out blind, it is possible that prisoners and staff may have changed their behaviour during data collection.
- Pre-policy samples were taken during the winter months and post-policy samples were taken during the summer months, greater ventilation post-policy may have contributed to the reduction in particulate matter.

For peer review only

INTRODUCTION

Since it was introduced a decade ago, smoke-free legislation in the United Kingdom (UK) has been successful in protecting the general public and workforce from harm arising from exposure to second-hand smoke (SHS) [1-3]. However the legislation included an exemption for Her Majesty's Prison and Probation Service (HMPPS, formally The National Offender Management Service (NOMS)) in England and Wales [4]. The exemption allowed aged over 18 years to smoke in a single cell or in a cell shared with other smokers [5], staff smoking was prohibited within prison perimeter walls. Since around 80% of UK prisoners are smokers [6-9], and many of these are highly tobacco dependent [9, 10], prisoners and prison staff remain at risk of high levels of SHS exposure.

In recent years, HMPPS have come under mounting pressure, from both the Prison Officers' Association (POA, the trade union representing prison officers throughout the UK) [11] and from legal challenges by non-smoking prisoners citing poor health due to personal frequent exposure to SHS [12], to implement a smoke-free policy throughout the prison estate in England and Wales. In September 2015, in response to empirical research demonstrating high levels of SHS in English prisons [13, 14], HMPPS announced the pilot implementation of a comprehensive smoke-free policy in four prisons in the South-West of England [15]. This policy prohibited all staff members and prisoners from possessing tobacco or smoking paraphernalia (such as lighters and cigarette rolling paper) within the perimeter walls of the four prisons.

Prior to implementation, smoking cessation services were offered to prisoners free of charge (this included behavioural support and pharmacotherapy), and disposable electronic cigarettes were made available to purchase through the prison canteen. Tobacco and smoking paraphernalia were removed from the canteen list two weeks before the smoke-free date at each establishment, to give prisoners the opportunity to smoke but not replace any remaining tobacco before the implementation date.

For peer review only

As part of an evaluation of the smoke-free policies introduced in four prisons in the South-West of England in 2016 we have compared indoor air quality, measured as concentrations of airborne particulate matter <2.5 microns in diameter ($PM_{2.5}$), on wing landing locations three months before and three months after going smoke-free. By measuring concentrations of $PM_{2.5}$, this study intends to determine to what extent the new policy reduces concentrations of SHS.

METHODS

Study prisons

Data were collected from the first four English Prison Service establishments selected to go smoke-free, with one prison going smoke-free every two weeks between 11th April and 23rd May 2016. The prisons were all in the South-West of England, and were selected for reasons including their low transfer rate to other regional areas, being all-male establishments, and having a relatively stable population. According to HMPPS annual performance ratings, all four prisons were performing well at the time of data collection and had reported no recent incidents [16]. One was a local prison (HMP 1) which served the courts and held both remand and convicted prisoners, while the other three were training prisons (HMPS 2, 3, & 4) which only held sentenced prisoners who are likely to be employed in day time activities (e.g. workshops or education). All four prisons had a Care and Separation Unit.

Before the smoke-free policy was implemented, all four prisons had a non-smoking policy for staff members within the perimeter wall, while prisoners were allowed to smoke only in their cells. However, although not permitted, prisoner smoking still occurred regularly on the exercise yards. The only exception prior to the smoke-free policy was the residential healthcare unit at HMP 1, which was designated ‘smoke-free’ and in which all indoor smoking was prohibited, to include cells. This unit was therefore excluded from our study.

For peer review only

Particulate pollution

The concentration of airborne particulate matter <2.5 microns in diameter ($PM_{2.5}$) is a well-established marker of indoor SHS concentrations [17, 18], and previous studies have shown high $PM_{2.5}$ concentrations in environments where smoking has taken place [17, 19]. Battery operated SidePak Personal Aerosol Monitors AM510 (TSI Inc, MN, USA) have been successfully used to measure $PM_{2.5}$ in prison environments previously [14, 20-22], as they are small, portable and do not require mains electricity (giving researchers the freedom over static placement on the wing landings). The SidePak uses a built-in sampling pump to draw air through the device, which then measures the concentration in milligrams per cubic metre of $PM_{2.5}$. The monitor logs $PM_{2.5}$ measurements at one minute intervals, with each one minute data point being an average of 60 one seconds sample measurements. Eleven SidePak Monitors fitted with impactor heads in order to measure $PM_{2.5}$ and set to a calibration factor of 0.30, as appropriate for tobacco smoke [23, 24] were used to measure $PM_{2.5}$ concentrations at each prison visit for this study. In accordance with manufacturer's instructions, SidePak devices were cleaned, the impactor re-greased, zero-calibrated and the flow rate set at 1.7 l/min before each use. Data were collected over three or four consecutive weekdays before the smoke-free policy was introduced, where possible, data collection was then repeated after the policy was introduced (repeating data collection at the same prison, day of the week, time of day, wing location and position of monitor). See Table 1 for study prison characteristics and data collection dates.

Two researchers trained in the use of air quality monitors placed the SidePak monitors in static locations on wing landings. Samples collected were compared with current World Health Organisation (WHO) indoor air quality standards, which recommend that $PM_{2.5}$ concentrations alone should not exceed 25 μg/m³ as a 24-hour mean [25].

Data collection

The four prisons were visited by two researchers three months before and after each prison's smoke-free implementation date (see Table 1). The two researchers were assigned a prison officer during their data collection to gain access to all the wing landings to place the SidePak air monitors in static locations, and to advise on areas of the prison that were not currently accessible for the researchers to visit (typically due to prisoner incidents). A wing landing is the communal shared area that all cell doors on a wing open onto, often housing showers, telephones and is typically a place where prisoners can spend time out of their cell during designated periods of the day. Pre-policy, researchers aimed to gain access to every prison landing at all four prisons at least once to sample $PM_{2.5}$ concentrations. Each air quality sample was identified with a unique code and data were recorded by a researcher on a sampling log sheet, to include; the prison; date and day of data collection; wing location and position of monitor; time the monitor was switched on and off; whether there was evidence that the monitor had been moved or tampered with; monitor serial number, and visit number (visit 1 = pre-policy implementation, visit 2= post-policy implementation). Typically, the two researchers were escorted around each prison twice a day, (morning and afternoon) in order to retrieve and place monitors in static locations. Researchers worked as a pair, with one completing the sampling log sheet whilst the other positioned or retrieved the monitors and checked if they had been tampered with or moved. Pre-implementation sampling logs and unique codes were used post-implementation to guide repeat data collection; where feasible placing SidePak monitors on the same day of the week, wing location, monitor position, start time and duration of sample. The sampling duration of each datasets was determined by access to wings locations via the prison escort and the machines battery life (around 11 hours). Monitors were programmed to turn off before the end of their battery life. The monitors were usually placed half way down the wing, above head height and away from open outside doors, windows, or cooking equipment. Where possible, monitors were sat in discreet static locations to avoid disrupting prisoners' normal behaviour. For security reasons, researchers advised the officers on each wing

For peer review only

how long they should expect the monitor to stay on the landing for and where each monitor had been placed.

As air quality monitors had been removed by prisoners during earlier sampling at HMP 2, all monitors were placed at one end of the unit next to or inside the wing office. Therefore, samples were not directly taken from the wing landings. Due to the landing design of several wings at HMP 3, air quality monitors had to be placed in a cupboard which inhibited air flow.

Patient and Public Involvement

There was no patients' or public involvement in this study.

For peer review only

Table 1. Study prison characteristics and data collection dates

Prison	HMP 1	HMP 2	HMP 3	HMP 4
Category and function*	Male<br>Category B<br>Local	Male<br>Category C<br>Training	Male<br>Category C<br>Training	Male<br>Category C<br>Training
Structural design	Built 1850s<br>Victorian radial design	Built early 1800s<br>Singular wings	Built 1974<br>Five two story living blocks and quick build wings	Built 1960s<br>Mix of triangular, T-shaped and quick build wings
Number of wings	7	7	9	9˜
Prisoner roll count (pre-implementation)	505	634	706	518˜
Sampling dates pre-smoke-free	19/01/16 – 23/01/16	08/02/16 – 11/02/16	15/02/16 – 18/02/16	29/02/16 – 02/03/16
Smoke-free implementation date	11/04/16	25/04/16	09/05/16	23/05/16
Sampling dates post-smoke-free	05/07/16 – 08/07/16	18/07/16 – 21/07/16	22/08/16 – 25/08/16	15/08/16 – 17/08/16

*Category B prisons hold prisoners for whom the very highest conditions of security are not necessary but for whom escape must be made very difficult.
*Category C prisons hold prisoners who cannot be trusted in open conditions but who do not have the resources and will to make a determined escape attempt.
*Local prisons serve the courts and receive remand and post-conviction prisoners prior to their allocation to other establishments.
*Training prisons hold sentenced prisoners who tend to be employed in a variety of activities such as prison workshops, gardens and education and in offending behaviour programme.
˜HMP 4 closed two wings (and transferred all prisoners located on these wings) between pre- and post- smoke-free data sampling dates.

For peer review only - http://bmjopen.bmj.com/site/about/guidelines.xhtml

Data analysis

Each dataset was downloaded from the SidePak device using the monitors recommended software (Trakpro 4.6.1) and imported into STATA 13, alongside its unique code. Datasets were then paired using its unique code (paired for prison, day of the week, wing and monitor position) and corresponding sample times paired (to the minute) to compare $PM_{2.5}$ concentrations pre- and post-implementation. Data from monitors that appeared to have been moved or tampered with, and those with no paired sample, were discarded. Descriptive statistics for all paired data and paired data by prison ID were generated; including mean, range, median, interquartile range, and the proportion of time the $PM_{2.5}$ concentration exceeded WHO 24- hour mean $PM_{2.5}$ upper limit of 25 $\mu g/m^3$ [25]. The percentage change of $PM_{2.5}$ concentrations was determined by comparing the mean and median $PM_{2.5}$ levels overall and in each prison before and after smoke-free. The Wilcoxon signed-rank test was used to assess statistical significance between pre- and post- implementation $PM_{2.5}$ concentrations in each establishment. To illustrate the sampled $PM_{2.5}$ distribution from each prison before and after implementation of smoke-free, boxplots were constructed. Although $PM_{2.5}$ data distributions were skewed, we present arithmetic mean figures throughout since these are used by the WHO to define their upper guidance limits [25].

Ethics approval

The University of Nottingham, Medical School Ethics Committee (G06062013 CHS EPH) approved this study, it was then subsequently approved by NOMS, National Research Committee (NRC) (Ref: 2013-202) in July 2014. Permission to enter all four prisons for data collection was sought from the Deputy Director of Public Sector Prisons and the Deputy Director of Custody for the South-West area. The Governors at each prison also agreed to the research being undertaken at their establishments.

For peer review only

RESULTS

A total of 200 datasets were collected from 32 wing landing locations throughout the four prisons. One SidePak monitor was destroyed during pre-implementation data collection, and on 12 occasions monitors were tampered with by prisoners (for example, by blocking the air inlet or turning off the monitor). The remaining 187 datasets included 113 collected before and 74 collected after policy implementation; the lower number after implementation arose primarily from restrictions on access to some prison wings. We therefore generated 74 paired sets of data for analysis (paired by prison, day of the week, time of the day, wing and monitor placement) which are presented in this paper. The 74 paired data sets were taken from across 29 wing landings (post-policy two wings at HMP 4 had been closed and one wing at HMP 3 could not be accessed by researchers due to security concerns), sampling particulate matter for an average of 5 hours and 8 minutes. Across all four prisons, monitors were placed on wing landings in the morning between 08:16 – 10:22 and in the afternoon between 14:38-18:00. (See Table 2 for individual prison break down of mean sampling times and monitor placements times). On sampling days both pre- and post-policy implementation all wings, apart from the Care and Separation Units, were at or near full capacity, with prisoner occupancy per wing ranging from 19 to 180.

Combined data from all four prisons, comparing $PM_{2.5}$ concentrations collected pre- and post-implementation

Mean $PM_{2.5}$ concentrations on wing landing locations before the introduction of smoke-free policy were 39 μg/m³, and 13 μg/m³ after introduction, representing a 66% reduction in mean $PM_{2.5}$ concentrations (mean difference 26 μg/m³, 95% Confidence Intervals 25 μg/m³ to 26 μg/m³); and a 69% reduction in median $PM_{2.5}$ concentrations (from 26 μg/m³ to 8 μg/m³). The mean $PM_{2.5}$ concentration pre- implementation exceeded the WHO 24- hour mean $PM_{2.5}$ upper limit of 25 μg/m³ [25], and continuously monitored levels were above this limit for more than half of all sampling time (see Table 2).

For peer review only

Table 2. Summary of sampled $PM_{2.5}$ concentrations combined and individually for four prisons pre- and post- smoke-free implementation.

	Combined prison data		HMP 1		HMP 2		HMP 3		HMP 4	
**Visit∞**	**1**	**2**	**1**	**2**	**1**	**2**	**1**	**2**	**1**	**2**
**Number of paired datasets (total paired sample time hr:min)**	74<br>380:20		20<br>97:57		14<br>70:57		22<br>125:50		18<br>85:36	
**Mean sample time (hr:min)**	5:08		4:54		5:04		5:43		4:45	
**Range of sampling start times (hr:min)**	AM: 08:16 – 10:22<br>PM: 14:38 – 18:00		AM: 08:16 - 09:02<br>PM: 14:57 - 18:00		AM: 08:52 - 09:22<br>PM: 15:39 – 15:54		AM: 08:42 – 09:57<br>PM: 14:38 - 16:14		AM: 09:08- 10:20<br>PM: 15:22- 16:29	
**Arithmetic mean (and 1 minute range) of $PM_{2.5}$ concentrations (µg/m³)**	39<br>(0-1359)	13<br>(0-3073)	66<br>(2-678)	14<br>(0-635)	13<br>(0-121)	6<br>(0-30)	35<br>(0-1359)	15<br>(2-227)	36<br>(1-1058)	17<br>(0-3073)
**Arithmetic mean percentage reduction from pre- to post-implementation**	66%		79%		50%		58%		54%	
**Median (and IQR) of $PM_{2.5}$ concentrations (µg/m³)**	26<br>(15-46)	8<br>(4-15)	42<br>(27-76)	8<br>(4-16)	11<br>(6-17)	6<br>(2-9)	27<br>(17-44)	9<br>(5-17)	29<br>(18-44.5)	8<br>(4-18)
**Median percentage reduction from pre- to post-implementation**	69%		81%		45%		67%		72%	
**Percentage of time above 25 µg/m³^**	51%	11%	77%	13%	7%	0%	53%	14%	56%	15%

∞ Visit number, 1 = pre- smoke-free policy implementation, 2 = post- smoke-free policy implementation

For peer review only

IQR = interquartile range

^ WHO 24- hour mean $PM_{2.5}$ upper limit of 25 μg/m³

For peer review only

Individual data from all four prisons, comparing $PM_{2.5}$ concentrations collected pre- and post-implementation

Data for the four prisons sampled (Table 2) demonstrate that all but HMP 2 had mean $PM_{2.5}$ concentrations above the WHO 24- hour mean upper limit pre-policy implementation, and all had mean post-policy concentrations below this limit [25]. All four prisons saw a statistically significant reduction in the $PM_{2.5}$ concentration pre-to post- smoke-free policy (median percentage reductions, HMP1 = 81%, HMP 2 = 45%, HMP 3 = 67%, HMP 4 = 72%, all four prisons, $p<0.001$). HMP 1, the local prison, had the highest mean and median $PM_{2.5}$ concentrations pre-policy, and the largest percentage reduction post- policy for these samples. Apart from HMP 2 (where monitors were not placed directly on the wing landings), the other three prisons lowered the time spent over the WHO 24- hour mean $PM_{2.5}$ upper limit from 53-77% to 13-15%. Figure 1 shows box plots of the distribution of $PM_{2.5}$ concentrations measured in each prison before and after the smoke-free policy. An example of the difference in $PM_{2.5}$ concentration profiles on a main residential wing at HMP 3, pre- and post-implementation, is presented in Figure 2.

For peer review only

Figure 1. Box plots of $PM_{2.5}$ distributions in each of the four prisons pre- and post-smoke-free implementation.

[INSERT FIGURE 1]

The horizontal line in each box represents the median value and the top and bottom of the box represent the 25th and 75th percentile, with the lines extending from the top and bottom of the boxes widening to the 5th and 95th percentile of the distribution. For ease of representation, Figure 1 does not show $PM_{2.5}$ concentrations over 500 μg/m³ (this only applies to samples taken from HMPs 1 & 4).

Figure 2. Concentrations of $PM_{2.5}$ sampled on a main residential wing at HMP 3 pre- and post-smoke-free implementation.

[INSERT FIGURE 2]

For peer review only

DISCUSSION

This is the first study to compare particulate pollution before and after the implementation of smoke-free policy in English prisons. The air quality measures, which used concentrations of $PM_{2.5}$ as a proxy for SHS, demonstrate that before the smoke-free policy was introduced $PM_{2.5}$ levels were well in excess of the WHO 24- hour mean $PM_{2.5}$ upper limit [25], with half of all sampling time over this recommended guidance level. After introduction of the smoke-free policy there was a substantial and statistically significant reduction in $PM_{2.5}$ concentrations, to below the WHO upper guidance limit of 25 µg/m³ per 24-hour. However the range of concentrations sampled suggest that prisoners were still smoking on occasions under the smoke-free policy.

Our air quality measurements were not carried out in blind fashion, because researchers were obliged to answer questions from staff members and prisoners who enquired about the monitoring. However, whilst it is possible that prisoners or staff changed their behaviour in response to being monitored, we think that is unlikely to have occurred to any appreciable degree over the course of our measurements. SHS is not the only source of indoor $PM_{2.5}$, which includes particulate matter released from sources such as open fires, toasters and microwaves. However, where toasters and microwaves were present on the wings, every effort was made to place the SidePak monitors as far away from these as possible. Safe locations for the SidePak monitors were limited, but researchers tried to collect data from all wings at each prison. Since security concerns and the design of the wings at HMP 2 and HMP 3 required us to place the Sidepak monitors in wing offices (not directly on the wing landing) and in cupboards on several of the landings (which inhibited air flow), these measures are likely to have underestimated the true $PM_{2.5}$ concentrations on these wing locations pre- and post- smoke-free policy. Nevertheless, reductions in $PM_{2.5}$ concentrations were still observed after policy implementation in the majority of these samples. Similar issues with placement of SidePak monitors on wing locations were described in work carried out in a New

For peer review only

Zealand prison, but that study also reported a significant reduction in $PM_{2.5}$ concentration after going smoke-free [22].

As an inevitable consequence of the smoke-free implementation dates in the four prisons, pre-policy air quality samples were taken during the winter months and post-policy during the summer months. It is possible that greater ventilation through open windows in the summer months may have contributed to the reduction in particulate levels between these two time points. However, to minimise this bias, SidePak monitors were placed towards the centre of the wings and away from any open windows during sampling. To examine whether outdoor air pollution (not only derived from SHS) could have contributed to indoor $PM_{2.5}$ concentrations, a study which measured concentrations of particulate matter in 15 Scottish prisons, compared its indoor $PM_{2.5}$ concentrations to outdoor measurements taken via the nearest static government monitoring station [21]. Unfortunately, for this study, the nearest static government monitors were a considerable distance away (mean, 47 kilometres) from the four prison sites sampled and were all placed in urban city centre locations (three of the four prisons sampled in this study were in remote rural locations). As $PM_{2.5}$ is not specific to SHS and can also arise from traffic and industrial air pollution, researchers felt the comparison for this study was not suitable.

Pre-policy, researchers were able to work around any prison incidents (e.g regimes changes, prisoner disturbances) in an attempt to sample all wing locations throughout the four prisons. Post-policy, researchers did not have the same flexibility as the sampling schedule was predetermined (in order to pair the samples for prison, day of the week, time, wing and monitor location) therefore fewer datasets were collected. We are unable to say whether this has significantly biased our findings. We recognise that our estimates of the proportion of time spent above the WHO $PM_{2.5}$ upper guidance limit of 25 $\mu g/m^3$ as a 24-hour mean are not truly representative because the maximum sampling time was determined by access to the wings, the battery life of the SidePak monitors used (around 11 hours), and only being able to place the monitors onto the wings during daytime hours. Since

For peer review only

smoking does not occur during sleep, particulate levels are likely to have been considerably lower during the night. However our data does give a very good estimation, in view of the large amount of paired data collected pre- and post- policy (over 15 days pre- and post- policy), of SHS pollution during times when non-smokers would be exposed during waking hours.

In an earlier air quality monitoring study (which included two of the pilot smoke-free sites sampled here), we measured $PM_{2.5}$ concentrations on wing landings where prisoners were permitted to smoke in their cells that were slightly higher than those three months prior to the smoke-free implementation in the present study (mean values 44 µg/m³ and 39 µg/m³ respectively) [14]. A possible explanation for this is that the majority of samples taken in the current study were carried out on days leading up to the weekly delivery of tobacco to prisoners from the prison shop (data in this study were collected Monday-Friday, with canteen delivery typically occurring on Fridays) when many prisoners are running out of tobacco, whereas the earlier study included samples taken at the weekend (after tobacco delivery). This earlier study reported that $PM_{2.5}$ concentrations were higher immediately after canteen delivery days [14]. It is also possible however that three months before going smoke-free, prisoners were already starting to reduce their tobacco consumption or had been on a smoking cessation course at the prison in light of the impending policy. Further validation of SHS levels recorded in this study pre-policy comes from two further air quality monitoring studies carried out in Scottish prisons prior to their smoke-free policy [13, 21] which produced similar pre-policy $PM_{2.5}$ concentrations.

Since 2005 the USA, New Zealand, Canada, and Australia have all implemented smoke-free policies in their correctional facilities. International air quality studies from New Zealand and the US have shown that comprehensive smoke-free polices are effective in substantially reducing SHS concentrations [20, 22, 26]. All of these studies used markers of SHS, respirable particulate matter (for example, particulate matter less than 2.5 µg/m³ ($PM_{2.5}$)) [20, 22] and airborne nicotine [26] to sample prison locations pre- and post- policy. The percentage reductions in $PM_{2.5}$ concentrations in our study were very similar to those recorded in these other countries [20, 22, 26].

For peer review only

Alongside reduced SHS concentrations, the potential health benefits of introducing a comprehensive smoke-free policy have been outlined in a study which examines the 10 years since the US implemented its smoking ban in prisons. This study found that prisons which implemented a smoke-free policy had a 9% reduction in smoking related deaths (particularly cardiovascular and pulmonary deaths), and that bans in place for longer than nine years were associated with a reduction in cancer deaths [27]. A study exploring natural deaths in male prisoners over 60 years of age in England and Wales reported diseases of the circulatory system (such as, coronary heart disease and cerebrovascular disease) and respiratory illnesses, all of which are substantially more common among smokers, as the most common cause of death [28]. With these findings in mind, the future roll-out of a comprehensive smoke-free policy across all 121 prisons in England and Wales set out by HMPPS [15] has the potential to have the same positive health impact on the nearly 83,000 prisoners [29] currently held, and the 32,000 staff members employed [30], in the prison estate.

Findings from this study suggest prisoners were still smoking after the introduction of smoke-free, since $PM_{2.5}$ concentrations post- policy ranged from 0-3073 $\mu g/m^3$, consistent with continued smoking in some areas. Hammond and colleagues measured nicotine concentrations before and after prisons in California, US went smoke-free, and concluded that a smoking ban was effective in reducing SHS exposure but did not eliminate it [26]. An ethnographic case study conducted in ten prisons in the US after implementing a complete smoking ban described the lengths prisoners would go to in order to acquire, exchange and smoke tobacco, and how tobacco had now become a more lucrative commodity to sell due to big demand and higher profit margin than illicit drugs [31]. The study concluded that although prisoners smoked less post-policy, the emergent black-market created by banning tobacco had a negative impact on inmates. The emergence of a tobacco black market was also observed in New Zealand and the Northern Territories of Australia after their implementation of smoke-free [31-33].

Conclusion

Smoke-free policies in these prisons has successfully reduced prisoner smoking, and both prisoner and staff exposure to SHS. Further work to reduce still further the occurrence of prisoner smoking is clearly required, and to assess the impact of the smoke-free policy on prisoner health. However, our data provide strong evidence in support of the continued implementation of the smoke-free policy throughout the English and Welsh prison estate and in other penal systems internationally.

For peer review only

Security clearance

Researchers (LJ & CH) were security cleared to Enhanced Level 1, enabling them to visit establishments and work within NOMS. Researchers were given security talks from each establishments prior to data collection.

Authors' contributors

LJ is the guarantor and takes responsibility for the integrity of the work as a whole, from inception to publication; study conception and design, data collection, analysis of data, interpretation of data and drafting the research manuscript. RM, ER and JB contributed to the study conception and design. CH assisted with data collection and MOB supported data preparation in STATA and data analysis. RM and JB aided drafting the manuscript. All authors read and approved the final manuscript.

Acknowledgements

The lead author would like to thank all four prisons for agreeing to participate in this research study, alongside the staff members who escorted the researchers during data collection.

Funding

This study was funded by a UK Centre for Tobacco and Alcohol Studies (UKCTAS) PhD studentship.

Data Sharing Statement

All available data can be obtained by contacting the corresponding author.

Competing Interests

None declared.

For peer review only

LEGEND

Figure 1. Box plots of $PM_{2.5}$ distributions in each of the four prisons pre- and post-smoke-free implementation.

Figure 2. Concentrations of $PM_{2.5}$ sampled on a main residential wing at HMP 3 pre- and post-smoke-free implementation.

For peer review only

REFERENCES

1. Bauld L. The impact of smokefree legislation in England: Evidence review. Available from: https://www.gov.uk/government/uploads/system/uploads/attachment_data/file/216319/dh_124959.pdf. 2011.

2. Department of Health. Smoke-free England - one year on. Available from: http://www.smokefreeengland.co.uk/files/dhs01_01-one-year-on-report-final.pdf London: UK. 2008.

3. Local Government Association. A breath of fresh air: Smokefree workplaces 10 years on. London: UK. June 2017

4. HM Government. Health Act 2006. Chapter 28, Smoking. Great Britain. 2006

5. Prison Service Instruction 09/2007. London: Smoke free legislation: prison service application. 2007.

6. Department of Health and HM Prison Service. Acquitted: Best practice guidance for developing smoking cessation services in prisons. London. 2003.

7. Lester C, Hamilton-Kirkwood L, and Jones N, Health indicators in a prison population: asking prisoners. Health Education Journal 2003;62:341-349.

8. Scottish Prison Service. Prisoner Survey 2015. Scottish Prison Service Scotland 2015.

9. Singleton, N., M. Farrell, and H. Meltzer. Substance misuse among prisoners in England and Wales. Available from: http://webarchive.nationalarchives.gov.uk/20160105160709/http://ons.gov.uk/ons/rel/psychiatric-morbidity/substance-misuse-among-prisoners/substance-misuse-among-prisoners/index.html. 1999.

10. Plugge EH, Foster CE, Yudkin PL, Douglas N, Cardiovascular disease risk factors and women prisoners in the UK: the impact of imprisonment. Health Promotion International. 2009; 24(4):334-343.

For peer review only

11. Prison Officers Association, Health and Safety Smoke Free. http://www.poauk.org.uk/index.php?circulars&newsdetail=20111114-185_health-safety-smoke-free-prison-estate. Prison Circular 2011.

12. Black -v- Secretary of State for Justice (Case No: CO/1258/2014): Hearing before the High Court Of Justice. 5 March 2015: Available from: https://www.judiciary.gov.uk/judgments/black-v-secretary-of-state-for-justice/.

13. Parsons Brinckerhoff. Report on Second-Hand Smoke in Prisons: Final Report. Available at - https://www.gov.uk/government/publications/air-quality-reports. 2015.

14. Jayes LR, Ratschen E, Murray RL, Dymond-White S, Britton J. Second-hand smoke in four English prisons: an air quality monitoring study. BMC Public Health. 2016;16(1):119.

15. Ministry of Justice. Ministry of Justice, Andrew Selous MP and National Offender Management Service. Letter from Prisons Minister Andrew Selous to Robert Neill MP, Chairman of the Justice Select Committee regarding smoking in prisons. September 2015.

16. The National Offender Management Service (NOMS). Prison Annual Performance Ratings 2015/16. Available from: https://www.gov.uk/government/uploads/system/uploads/attachment_data/file/541173/prison-annual-performance-ratings-2015-2016.pdf.

17. Hyland A, Travers MJ, and Dresler C. A 32-country comparison of tobacco smoke derived particle levels in indoor public places. Tobacco Control. Jun 2008;17(3):159-165.

18. Sureda X,Fernandez E, and Lopez MJ. Secondhand tobacco smoke exposure in open and semi-open settings: a systematic review. Environ Health Perspect. Jul 2013;121(7):766-73.

19. Semple S, Apsley A, Galea KS, MacCalman L, Friel B, Snelgrive V. Secondhand smoke in cars: assessing children's potential exposure during typical journey conditions. Tobacco Control. Nov 2012;(6):578-83.

20. Proescholdbell SK, Foley KL, and Johnson J. Indoor air quality in prisons before and after implementation of a smoking ban law. Tobacco Control. Apr 2008;(2):123-7.

21. Semple S, Sweeting H, Demou E, Logan G, O'Donnell R, Hunt K. Characterising the Exposure of Prison Staff to Second-Hand Tobacco Smoke. Annals of Work Exposures and Health. 2017;61(7):809-821.

22. Thornley S, Dirks KN, Edwards R. Indoor air pollution levels were halved as a result of a national tobacco ban in a New Zealand prison. Nicotine Tob Res. Feb 2013;15(2):343-7.

23. Jiang RT, Acevedo-Bolton V, and Cheng KC. Determination of response of real-time SidePak AM510 monitor to secondhand smoke, other common indoor aerosols, and outdoor aerosol. J Environ Monit. Jun 2011;13(6):1695-702.

24. Semple S, Tongeren M, and Galea KS. UK smoke-free legislation: changes in PM2.5 concentrations in bars in Scotland, England, and Wales. Ann Occup Hyg. Apr 2010;54(3):272-80.

25. WHO air quality guidelines. Global Update. Geneva, Switzerland. 2005.

26. Hammond SK, and Emmons KM. Inmate exposure to secondhand smoke in correctional facilities and the impact of smoking restrictions. J Expo Anal Environ Epidemiol. 2004;15(3):205-211.

27. Binswanger I, Carson E, Krueger P, Mueller S, Steiner J, Sabol W. Prison tobacco control policies and deaths from smoking in United States prisons: population based retrospective analysis. BMJ. 2014;349:g4542.

28. Fazel S, and Benning R. Natural deaths in male prisoners: a 20-year mortality study. European Journal of Public Health. 2006;16(4):441-444.

29. Ministry of Justice. National Offender Management Service (NOMS), Prison population figures: 2018. Available from: https://www.gov.uk/government/statistics/prison-population-figures-2018. 2018.

30. Ministry of Justice. Her Majesty's Prison and Probation Service workforce quarterly: June 2017, Workforce Statistics Bulletin, 2017: Available from: https://www.gov.uk/government/statistics/her-majestys-prison-and-probation-service-workforce-quarterly-june-2017. 2017.

For peer review only

31. Lankenau SE. Smoke 'em if you got 'em: Cigarette black markets in U.S. prisons and jails. The Prison journal. 2001;81(2):142-161.

32. Collinson L, Wilson N, Edwards R, Thomson G, Thornley S. New Zealand's smokefree prison policy appears to be working well: one year on. The New Zealand Medical Journal. 2012; 125(1357):164-168.

33. Hefler MR, Hopkins R, and Thomas D. Successes and unintended consequences of the Northern Territory's smoke-free prisons policy: results from a process evaluation. Public Health Research & Practice. Apr 2016;26(2)e26216219.

For peer review only

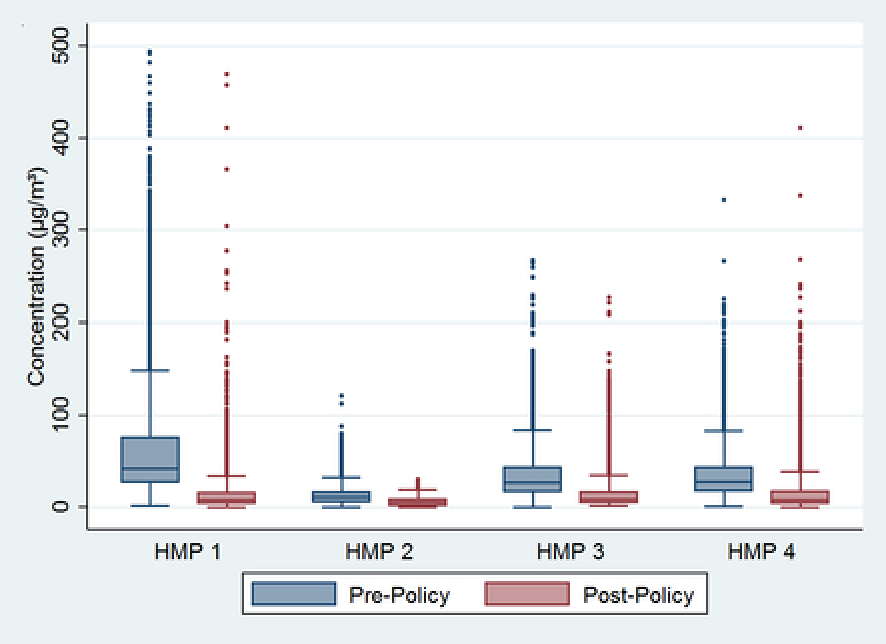

37x27mm (300 x 300 DPI)

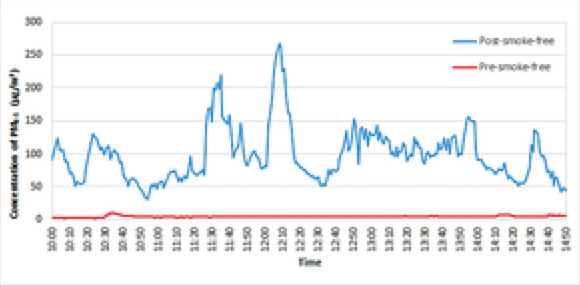

24x12mm (300 x 300 DPI)

STROBE checklist

Section	Page number	Explanatory Notes
**Title**	Pg. 1	ENTER TITLE ONCE DECIDED
**Abstract**	Pg. 2/3	Please refer to abstract section of the manuscript
**Introduction**		
Background	Pg.3/4	Refer to the 'Introduction' section of the manuscript for a brief background of the topic
Objectives	Pg.3/4	By measuring concentrations of airborne particulate matter <2.5 microns in diameter ($PM_{2.5}$) as a proxy for second-hand smoke (SHS), this study intends to determine to what extent a new smoke-free policy reduces concentrations of SHS in the first four prisons to go smoke-free in England.
**Methods**		
Study design	Pg. 1/2/5	Air quality monitoring study. In the title of the study and section 'design' in Abstract
Setting	Pg. 5/9	Great Britain, four prisons in the South-West of England. In 'Study prison' and Table 1 provides further information on each prison and outlines dates of data collection.
Participants	N/A	Our interest is in particulate matter pre- to post- smoke-free policy.
Variables	Pg. 6	Section 'particulate pollution' in Methods
Data source	Pg. 5/6	Section 'study prisons and particulate pollution' in Methods
Bias	Pg. 10/17	Section 'data analyses' in Methods and in Discussion section. Air quality measurements were not carried out blind and seasonal differences may have also impacted on the sample concentrations (winter v. summer). Where possible, placed monitors away from other sources of $PM_{2.5}$.
Study Size	Pg. 2/5	In 'abstract', 'introduction' and 'study prisons' sections. The prison service decision to pilot four smoke-free sites, all were sampled.
Quantitative variables	Pg. 10	Section 'data analysis' in Methods. Pre and post particulate matter were matched for prison, wing location and monitor placement, day of the week and time.
Statistical method	Pg. 10	Section 'data analysis' in Methods
**Results**		
Participants	N/A	Our interest is concentrations of airborne particulate matter <2.5 microns in diameter ($PM_{2.5}$) measured as a proxy for second-hand smoke (SHS).
Descriptive data	Pg.	Section 'results'- 74 paired sets of data collected over 29 wing locations in the four prisons sampled. Sampling $PM_{2.5}$ for an average of 5 hours and 8 minutes.
Outcome data	Pg. 11	Section 'combined data for all four prisons, comparing PM2.5 concentrations collected pre-and post-implementation'
Main Results	Pg. 11	Section 'combined data for all four prisons, comparing PM2.5 concentrations collected pre-and post-implementation'. Mean $PM_{2.5}$ concentrations on wing landing locations before the introduction of smoke-free policy were 39 μg/m³, and 13 μg/m³ after introduction, representing a 66% reduction in mean $PM_{2.5}$ concentrations (mean difference 26 μg/m³, 95% Confidence Intervals 25

		μg/m³ to 26 μg/m³); and a 69% reduction in median $PM_{2.5}$ concentrations (from 26 μg/m³ to 8 μg/m³).
Other analysis	Pg. 11/14	Results section 'combined data for all four prisons, comparing PM2.5 concentrations collected pre-and post-implementation' provide comparisons to WHO guidance levels and 'individual data from all four prisons, comparing $PM_{2.5}$ concentrations collected pre-and post-implementation' provides breakdown of results per prison.
**Discussion**		
Key Results	Pg. 16	First paragraph of the Discussion section.
Limitations	Pg.16/17/18	Paragraphs 2-4 in Discussion section.
Interpretation	Pg. 18	Paragraph 4 in Discussion - our data does give a very good estimation, in view of the large amount of matched data collected pre- and post- policy (over 15 days pre- and post-policy), of SHS pollution during times when non-smokers would be exposed during waking hours.
Generalisability	Pg. 20	Paragraph 6 and 'conclusion' in Discussion
**Other Information**		
Funding	Pg. 21	This study was funded by a UK Centre for Tobacco and Alcohol Studies (UKCTAS) PhD studentship.

For peer review only

BMJ Open

Smoke-free prisons in England: Indoor air quality before and after implementation of a comprehensive smoke-free policy

Journal:	*BMJ Open*
Manuscript ID	bmjopen-2018-025782.R2
Article Type:	Research
Date Submitted by the Author:	11-Feb-2019
Complete List of Authors:	Jayes, Leah; The University of Nottingham , Medicine<br>Murray, Rachael; The University of Nottingham , Medicine<br>Opazo Breton, Magdalena; University of Nottingham, Epidemiology and Public Health<br>Hill , Christopher; University of Nottingham School of Medicine, Medicine<br>Ratschen, Elena; University of York, Health Sciences<br>Britton, John; The University of Nottingham
**Primary Subject Heading**:	Smoking and tobacco
Secondary Subject Heading:	Public health
Keywords:	PUBLIC HEALTH, EPIDEMIOLOGY, Epidemiology < ONCOLOGY

SCHOLARONE™
Manuscripts

Smoke-free prisons in England: Indoor air quality before and after implementation of a comprehensive smoke-free policy

Leah R Jayes[1], Rachael L Murray[1], Magdalena Opazo Breton[1], Christopher Hill[1], Elena Ratschen[2], John Britton[1]

[1]UK Centre for Tobacco and Alcohol Studies and Division of Epidemiology and Public Health, University of Nottingham, Clinical Sciences Building, Nottingham City Hospital, Nottingham, NG5 1PB, UK

[2]Department of Health Sciences, Mental Health and Addictions Research Group, Department of Health Sciences, University of York, York, YO10 5DD, UK

Correspondence to: LR Jayes. leah.jayes@nottingham.ac.uk / +44 115 8231367

For peer review only

Word Count: 4038

Number of Figures: 2

Number of Tables: 2

References: 33

Supplementary Figures: 0

ABSTRACT

Objectives

High levels of particulate pollution due to second-hand smoke (SHS) have previously been recorded in English prisons. As part of an evaluation to ascertain whether a new comprehensive smoke-free policy introduced in the first four prisons in England was successfully implemented, this study compares indoor air quality on prison wing landing locations three months before and three months after going smoke-free.

Design

An indoor air quality monitoring study, comparing SHS levels before and after a comprehensive smoke-free prison policy.

Setting

The first four prisons in England to implement a comprehensive smoke-free policy.

Primary and secondary measures

We compared concentrations of airborne particulate matter <2.5 microns in diameter ($PM_{2.5}$), as a marker for SHS, on wing landing locations three months before and three months after the smoke-free policy was implemented. Static battery operated aerosol monitors were used to sample concentrations of $PM_{2.5}$ on wing landings.

Results

After discarding data from monitors that had been tampered with we were able to analyse paired data across four prisons from 74 locations, across 29 wing landing locations, for an average sampling time of five hours and eight minutes. When comparing samples taken three months before with the paired samples taken three months after policy implementation (paired for prison, day of the week, time of day, wing location and position of monitor), there was a statistically significant ($p<0.001$)

For peer review only

66% reduction in mean $PM_{2.5}$ concentrations at each of the four prisons sampled, from 39 µg/m³ to 13µg/m³ (difference 26 µg/m³, 95% CI 25 µg/m³ to 26 µg/m³).

Conclusion

Prison smoke-free policies achieve significant improvements in indoor air quality. A national smoke-free policy would therefore be an effective means of protecting prisoners and staff from harm due to SHS exposure in the prison environment.

Strengths and limitations of this study

- This is the first study to compare particulate pollution before and after the implementation of a smoke-free policy in English prisons.
- Air quality monitoring was not carried out blind, it is possible that prisoners and staff may have changed their behaviour during data collection.
- Pre-policy samples were taken during the winter months and post-policy samples were taken during the summer months, greater ventilation post-policy may have contributed to the reduction in particulate matter.

For peer review only

INTRODUCTION

Since it was introduced a decade ago, smoke-free legislation in the United Kingdom (UK) has been successful in protecting the general public and workforce from harm arising from exposure to second-hand smoke (SHS) [1-3]. However the legislation included an exemption for Her Majesty's Prison and Probation Service (HMPPS, formally The National Offender Management Service (NOMS)) in England and Wales [4]. The exemption allowed prisoners aged over 18 years to smoke in a single cell or in a cell shared with other smokers [5], staff smoking was prohibited within prison perimeter walls. Since around 80% of UK prisoners are smokers [6-9], and many of these are highly tobacco dependent [9, 10], prisoners and prison staff remain at risk of high levels of SHS exposure.

In recent years, HMPPS have come under mounting pressure, from both the Prison Officers' Association (POA, the trade union representing prison officers throughout the UK) [11] and from legal challenges by non-smoking prisoners citing poor health due to personal frequent exposure to SHS [12], to implement a smoke-free policy throughout the prison estate in England and Wales. In September 2015, in response to empirical research demonstrating high levels of SHS in English prisons [13, 14], HMPPS announced the pilot implementation of a comprehensive smoke-free policy in four prisons in the South-West of England [15]. This policy prohibited all staff members and prisoners from smoking tobacco and possessing tobacco or smoking paraphernalia (such as lighters and cigarette rolling paper) within the perimeter walls of the four prisons.

Prior to implementation, smoking cessation services were offered to prisoners free of charge (this included behavioural support and pharmacotherapy), and disposable electronic cigarettes were made available to purchase through the prison canteen. Prisoners were only permitted to vape whilst in their cell. Tobacco and smoking paraphernalia were removed from the canteen list two weeks before the smoke-free date at each establishment, to give prisoners the opportunity to smoke but not replace any remaining tobacco before the implementation date.

For peer review only

As part of an evaluation of the smoke-free policies introduced in four prisons in the South-West of England in 2016 we have compared indoor air quality, measured as concentrations of airborne particulate matter <2.5 microns in diameter ($PM_{2.5}$), on wing landing locations three months before and three months after going smoke-free. By measuring concentrations of $PM_{2.5}$, this study intends to determine to what extent the new policy reduces concentrations of SHS.

METHODS

Study prisons

Data were collected from the first four English Prison Service establishments selected to go smoke-free, with one prison going smoke-free every two weeks between 11th April and 23rd May 2016. The prisons were all in the South-West of England, and were selected for reasons including their low transfer rate to other regional areas, being all-male establishments, and having a relatively stable population. According to HMPPS annual performance ratings, all four prisons were performing well at the time of data collection and had reported no recent incidents [16]. One was a local prison (HMP 1) which served the courts and held both remand and convicted prisoners, while the other three were training prisons (HMPS 2, 3, & 4) which only held sentenced prisoners who are likely to be employed in day time activities (e.g. workshops or education). All four prisons had a Care and Separation Unit.

Before the smoke-free policy was implemented, all four prisons had a non-smoking policy for staff members within the perimeter wall, while prisoners were allowed to smoke only in their cells. However, although not permitted, prisoner smoking still occurred regularly on the exercise yards. The only exception prior to the smoke-free policy was the residential healthcare unit at HMP 1, which was designated 'smoke-free' and in which all indoor smoking was prohibited, to include cells. This unit was therefore excluded from our study.

For peer review only

Particulate pollution

The concentration of airborne particulate matter <2.5 microns in diameter ($PM_{2.5}$) is a well-established marker of indoor SHS concentrations [17, 18], and previous studies have shown high $PM_{2.5}$ concentrations in environments where smoking has taken place [17, 19]. Battery operated SidePak Personal Aerosol Monitors AM510 (TSI Inc, MN, USA) have been successfully used to measure $PM_{2.5}$ in prison environments previously [14, 20-22], as they are small, portable and do not require mains electricity (giving researchers the freedom over static placement on the wing landings). The SidePak uses a built-in sampling pump to draw air through the device, which then measures the concentration in milligrams per cubic metre of $PM_{2.5}$. The monitor logs $PM_{2.5}$ measurements at one minute intervals, with each one minute data point being an average of 60 one second sample measurements. Eleven SidePak Monitors fitted with impactor heads in order to measure $PM_{2.5}$ and set to a calibration factor of 0.30, as appropriate for tobacco smoke [23, 24] were used to measure $PM_{2.5}$ concentrations at each prison visit for this study. In accordance with manufacturer's instructions, SidePak devices were cleaned, the impactor re-greased, zero-calibrated and the flow rate set at 1.7 l/min before each use. Data were collected over three or four consecutive weekdays before the smoke-free policy was introduced. Where possible, data collection was then repeated after the policy was introduced (repeating data collection at the same prison, day of the week, time of day, wing location and position of monitor). See Table 1 for study prison characteristics and data collection dates.

Two researchers trained in the use of air quality monitors placed the SidePak monitors in static locations on wing landings. Samples collected were compared with current World Health Organisation (WHO) indoor air quality standards, which recommend that $PM_{2.5}$ concentrations alone should not exceed 25 $\mu g/m^3$ as a 24-hour mean [25].

For peer review only - http://bmjopen.bmj.com/site/about/guidelines.xhtml

Data collection

The four prisons were visited by two researchers three months before and after each prison's smoke-free implementation date (see Table 1). The two researchers were assigned a prison officer during their data collection to gain access to all the wing landings to place the SidePak air monitors in static locations, and to advise on areas of the prison that were not currently accessible for the researchers to visit (typically due to prisoner incidents). A wing landing is the communal shared area that all cell doors on a wing open onto, often housing showers, telephones and is typically a place where prisoners can spend time out of their cell during designated periods of the day. Pre-policy, researchers aimed to gain access to every prison landing at all four prisons at least once to sample $PM_{2.5}$ concentrations. Each air quality sample was identified with a unique code and data were recorded by a researcher on a sampling log sheet, to include; the prison; date and day of data collection; wing location and position of monitor; time the monitor was switched on and off; whether there was evidence that the monitor had been moved or tampered with; monitor serial number, and visit number (visit 1 = pre-policy implementation, visit 2= post-policy implementation). Typically, the two researchers were escorted around each prison twice a day, (morning and afternoon) in order to retrieve and place monitors in static locations. Researchers worked as a pair, with one completing the sampling log sheet whilst the other positioned or retrieved the monitors and checked if they had been tampered with or moved. Pre-implementation sampling logs and unique codes were used post-implementation to guide repeat data collection; where feasible placing SidePak monitors on the same day of the week, wing location, monitor position, start time and duration of sample. The sampling duration of each dataset was determined by access to wings locations via the prison escort and the machine's battery life (around 11 hours). Monitors were programmed to turn off before the end of their battery life. The monitors were usually placed half way down the wing, above head height and away from open outside doors, windows, or cooking equipment. Where possible, monitors were placed in discreet static locations to avoid disrupting prisoners' normal behaviour. For security reasons, researchers advised the officers on each wing

how long they should expect the monitor to stay on the landing for and where each monitor had been placed.

As air quality monitors had been removed by prisoners during earlier sampling at HMP 2, all monitors in this prison were placed at one end of the unit next to or inside the wing office. Therefore, samples were not directly taken from the wing landings. Due to the landing design of several wings at HMP 3, air quality monitors had to be placed in a cupboard which inhibited air flow.

Patient and Public Involvement

There was no patient or public involvement in this study.

For peer review only

Table 1. Study prison characteristics and data collection dates

Prison	HMP 1	HMP 2	HMP 3	HMP 4
Category and function*	Male<br>Category B<br>Local	Male<br>Category C<br>Training	Male<br>Category C<br>Training	Male<br>Category C<br>Training
Structural design	Built 1850s<br>Victorian radial design	Built early 1800s<br>Singular wings	Built 1974<br>Five two story living blocks and quick build wings	Built 1960s<br>Mix of triangular, T-shaped and quick build wings
Number of wings	7	7	9	9˜
Smoke-free implementation date	11/04/16	25/04/16	09/05/16	23/05/16
Prisoner roll count pre-policy	505	634	706	518
Prisoner roll count post-policy	477	628	691	378˜
Sampling dates pre-policy	19/01/16 – 23/01/16	08/02/16 – 11/02/16	15/02/16 – 18/02/16	29/02/16 – 02/03/16
Sampling dates post-policy	05/07/16 – 08/07/16	18/07/16 – 21/07/16	22/08/16 – 25/08/16	15/08/16 – 17/08/16

*Category B prisons hold prisoners for whom the very highest conditions of security are not necessary but for whom escape must be made very difficult.
*Category C prisons hold prisoners who cannot be trusted in open conditions but who do not have the resources and will to make a determined escape attempt.
*Local prisons serve the courts and receive remand and post-conviction prisoners prior to their allocation to other establishments.
*Training prisons hold sentenced prisoners who tend to be employed in a variety of activities such as prison workshops, gardens and education and in offending behaviour programme.
˜HMP 4 closed two wings (and transferred all prisoners located on these wings) between pre- and post- smoke-free data sampling dates.

For peer review only

Data analysis

Each dataset was downloaded from the SidePak device using the monitor's recommended software (Trakpro 4.6.1) and imported into STATA 13, alongside its unique code. Datasets were then paired using their unique code (paired for prison, day of the week, wing and monitor position) and corresponding sample times paired (to the minute) to compare $PM_{2.5}$ concentrations pre- and post-implementation. Data from monitors that appeared to have been moved or tampered with, and those with no paired sample, were discarded. Descriptive statistics for all paired data and paired data by prison ID were generated; including mean, range, median, interquartile range, and the proportion of time the $PM_{2.5}$ concentration exceeded WHO 24- hour mean $PM_{2.5}$ upper limit of 25 $\mu g/m^3$ [25]. The percentage change of $PM_{2.5}$ concentrations was determined by comparing the mean and median $PM_{2.5}$ levels overall and in each prison before and after smoke-free. The Wilcoxon signed-rank test was used to assess statistical significance between pre- and post- implementation $PM_{2.5}$ concentrations in each establishment. To illustrate the sampled $PM_{2.5}$ distribution from each prison before and after implementation of smoke-free, boxplots were constructed. Although $PM_{2.5}$ data distributions were skewed, we present arithmetic mean figures throughout since these are used by the WHO to define their upper guidance limits [25].

Ethics approval

The University of Nottingham, Medical School Ethics Committee (G06062013 CHS EPH) approved this study, it was then subsequently approved by NOMS, National Research Committee (NRC) (Ref: 2013-202) in July 2014. Permission to enter all four prisons for data collection was sought from the Deputy Director of Public Sector Prisons and the Deputy Director of Custody for the South-West area. The Governors at each prison also agreed to the research being undertaken at their establishments.

For peer review only

RESULTS

A total of 200 datasets were collected from 32 wing landing locations throughout the four prisons. One SidePak monitor was destroyed during pre-implementation data collection, and on 12 occasions monitors were tampered with by prisoners (for example, by blocking the air inlet or turning off the monitor). The remaining 187 datasets included 113 collected before and 74 collected after policy implementation; the lower number after implementation arose primarily from restrictions on access to some prison wings. We therefore generated 74 paired sets of data for analysis (paired by prison, day of the week, time of the day, wing and monitor placement) which are presented in this paper. The 74 paired data sets were taken from across 29 wing landings (post-policy two wings at HMP 4 had been closed and one wing at HMP 3 could not be accessed by researchers due to security concerns), sampling particulate matter for an average of 5 hours and 8 minutes. Across all four prisons, monitors were placed on wing landings in the morning between 08:16 – 10:22 and in the afternoon between 14:38-18:00. (See Table 2 for individual prison break down of mean sampling times and monitor placements times). On sampling days both pre- and post-policy implementation, all wings (apart from the Care and Separation Units) were at or near full capacity, with prisoner occupancy per wing ranging from 19 to 180.

Combined data from all four prisons, comparing $PM_{2.5}$ concentrations collected pre- and post-implementation

Mean $PM_{2.5}$ concentrations on wing landing locations before the introduction of smoke-free policy were 39 µg/m³, and 13 µg/m³ after introduction, representing a 66% reduction in mean $PM_{2.5}$ concentrations (mean difference 26 µg/m³, 95% Confidence Intervals 25 µg/m³ to 26 µg/m³); and a 69% reduction in median $PM_{2.5}$ concentrations (from 26 µg/m³ to 8 µg/m³). The mean $PM_{2.5}$ concentration pre- implementation exceeded the WHO 24- hour mean $PM_{2.5}$ upper limit of 25 µg/m³ [25], and continuously monitored levels were above this limit for half of all sampling time (see Table 2).

For peer review only

Table 2. Summary of sampled $PM_{2.5}$ concentrations combined and individually for four prisons pre- and post- smoke-free implementation.

	Combined prison data		HMP 1		HMP 2		HMP 3		HMP 4	
**Visit∞**	**1**	**2**	**1**	**2**	**1**	**2**	**1**	**2**	**1**	**2**
**Number of paired datasets (total paired sample time hr:min)**	74<br>380:20		20<br>97:57		14<br>70:57		22<br>125:50		18<br>85:36	
**Mean sample time (hr:min)**	5:08		4:54		5:04		5:43		4:45	
**Range of sampling start times (hr:min)**	AM: 08:16 – 10:22<br>PM: 14:38 – 18:00		AM: 08:16 - 09:02<br>PM: 14:57 - 18:00		AM: 08:52 - 09:22<br>PM: 15:39 – 15:54		AM: 08:42 – 09:57<br>PM: 14:38 - 16:14		AM: 09:08- 10:20<br>PM: 15:22- 16:29	
**Arithmetic mean (and 1 minute range) of $PM_{2.5}$ concentrations (µg/m³)**	39<br>(0-1359)	13<br>(0-3073)	66<br>(2-678)	14<br>(0-635)	13<br>(0-121)	6<br>(0-30)	35<br>(0-1359)	15<br>(2-227)	36<br>(1-1058)	17<br>(0-3073)
**Arithmetic mean percentage reduction from pre- to post-implementation**	66%		79%		50%		58%		54%	
**Median (and IQR) of $PM_{2.5}$ concentrations (µg/m³)**	26<br>(15-46)	8<br>(4-15)	42<br>(27-76)	8<br>(4-16)	11<br>(6-17)	6<br>(2-9)	27<br>(17-44)	9<br>(5-17)	29<br>(18-44.5)	8<br>(4-18)
**Median percentage reduction from pre- to post-implementation**	69%		81%		45%		67%		72%	
**Percentage of time above 25 µg/m³^**	51%	11%	77%	13%	7%	0%	53%	14%	56%	15%

∞ Visit number, 1 = pre- smoke-free policy implementation, 2 = post- smoke-free policy implementation

For peer review only

IQR = interquartile range

^ WHO 24- hour mean $PM_{2.5}$ upper limit of 25 µg/m^3

For peer review only

Individual data from all four prisons, comparing $PM_{2.5}$ concentrations collected pre- and post-implementation

Data for the four prisons sampled (Table 2) demonstrate that all but HMP 2 had mean $PM_{2.5}$ concentrations above the WHO 24- hour mean upper limit pre-policy implementation, and all had mean post-policy concentrations below this limit [25]. All four prisons saw a statistically significant reduction in the $PM_{2.5}$ concentration pre-to post- smoke-free policy (median percentage reductions, HMP1 = 81%, HMP 2 = 45%, HMP 3 = 67%, HMP 4 = 72%, all four prisons, $p<0.001$). HMP 1, the local prison, had the highest mean and median $PM_{2.5}$ concentrations pre-policy, and the largest percentage reduction post- policy for these samples. In HMP 2 (where monitors were not placed directly on the wing landings) the time spent over the WHO 24- hour mean $PM_{2.5}$ upper limit reduced from 7% to 0%; in the other three prisons, the reduction was from 53-77% pre- to 13-15% post- policy implementation. Figure 1 shows box plots of the distribution of $PM_{2.5}$ concentrations measured in each prison before and after the smoke-free policy. An example of the difference in $PM_{2.5}$ concentration profiles on a main residential wing at HMP 3, pre- and post-implementation, is presented in Figure 2.

For peer review only

Figure 1. Box plots of $PM_{2.5}$ distributions in each of the four prisons pre- and post-smoke-free implementation.

[INSERT FIGURE 1]

The horizontal line in each box represents the median value and the top and bottom of the box represent the 25th and 75th percentile, with the lines extending from the top and bottom of the boxes widening to the 5th and 95th percentile of the distribution. For ease of representation, Figure 1 does not show $PM_{2.5}$ concentrations over 500 µg/m^3 (this only applies to samples taken from HMPs 1 & 4).

Figure 2. Concentrations of $PM_{2.5}$ sampled on a main residential wing at HMP 3 pre- and post-smoke-free implementation.

[INSERT FIGURE 2]

For peer review only

DISCUSSION

This is the first study to compare particulate pollution before and after the implementation of smoke-free policy in English prisons. The air quality measures, which used concentrations of $PM_{2.5}$ as a proxy for SHS, demonstrate that before the smoke-free policy was introduced $PM_{2.5}$ levels were well in excess of the WHO 24- hour mean $PM_{2.5}$ upper limit [25], with half of all sampling time over this recommended guidance level. After introduction of the smoke-free policy there was a substantial and statistically significant reduction in $PM_{2.5}$ concentrations, to below the WHO upper guidance limit of 25 μg/m³ per 24-hour. However the range of concentrations sampled suggest that prisoners were still smoking on occasions under the smoke-free policy.

Our air quality measurements were not carried out in blind fashion, because researchers were obliged to answer questions from staff members and prisoners who enquired about the monitoring. However, whilst it is possible that prisoners or staff changed their behaviour in response to being monitored, we think that is unlikely to have occurred to any appreciable degree over the course of our measurements. SHS is not the only source of indoor $PM_{2.5}$, which includes particulate matter released from sources such as open fires, toasters and microwaves. However, where toasters and microwaves were present on the wings, every effort was made to place the SidePak monitors as far away from these as possible. Safe locations for the SidePak monitors were limited, but researchers tried to collect data from all wings at each prison. Since security concerns and the design of the wings at HMP 2 and HMP 3 required us to place the Sidepak monitors in wing offices (not directly on the wing landing) and in cupboards on several of the landings (which inhibited air flow), these measures are likely to have underestimated the true $PM_{2.5}$ concentrations on these wing locations pre- and post- smoke-free policy. Nevertheless, reductions in $PM_{2.5}$ concentrations were still observed after policy implementation in the majority of these samples. Similar issues with placement of SidePak monitors on wing locations were described in work carried out in a New

For peer review only

Zealand prison, but that study also reported a significant reduction in $PM_{2.5}$ concentration after going smoke-free [22].

As an inevitable consequence of the smoke-free implementation dates in the four prisons, pre-policy air quality samples were taken during the winter months and post-policy during the summer months. It is possible that greater ventilation through open windows in the summer months may have contributed to the reduction in particulate levels between these two time points. However, to minimise this bias, SidePak monitors were placed towards the centre of the wings and away from any open windows during sampling. To examine whether outdoor air pollution (not only derived from SHS) could have contributed to indoor $PM_{2.5}$ concentrations, a study which measured concentrations of particulate matter in 15 Scottish prisons, compared its indoor $PM_{2.5}$ concentrations to outdoor measurements taken via the nearest static government monitoring station [21]. Unfortunately, for this study, the nearest static government monitors were a considerable distance away (mean, 47 kilometres) from the four prison sites sampled and were all placed in urban city centre locations (three of the four prisons sampled in this study were in remote rural locations). As $PM_{2.5}$ is not specific to SHS and can also arise from traffic and industrial air pollution, researchers felt the comparison for this study was not suitable.

Pre-policy, researchers were able to work around any prison incidents (e.g regimes changes, prisoner disturbances) in an attempt to sample all wing locations throughout the four prisons. Post-policy, researchers did not have the same flexibility as the sampling schedule was predetermined (in order to pair the samples for prison, day of the week, time, wing and monitor location) therefore fewer datasets were collected. We are unable to say whether this has significantly biased our findings. We recognise that our estimates of the proportion of time spent above the WHO $PM_{2.5}$ upper guidance limit of 25 μg/m³ as a 24-hour mean are not truly representative because the maximum sampling time was determined by access to the wings, the battery life of the SidePak monitors used (around 11 hours), and only being able to place the monitors onto the wings during daytime hours. Since

For peer review only

smoking does not occur during sleep, particulate levels are likely to have been considerably lower during the night. However our data does give a very good estimation, in view of the large amount of paired data collected pre- and post- policy (over 15 days pre- and post- policy), of SHS pollution during times when non-smokers would be exposed during waking hours.

In an earlier air quality monitoring study (which included two of the pilot smoke-free sites sampled here), we measured $PM_{2.5}$ concentrations on wing landings where prisoners were permitted to smoke in their cells that were slightly higher than those three months prior to the smoke-free implementation in the present study (mean values 44 µg/m³ and 39 µg/m³ respectively) [14]. A possible explanation for this is that the majority of samples taken in the current study were carried out on days leading up to the weekly delivery of tobacco to prisoners from the prison shop (data in this study were collected Monday-Friday, with canteen delivery typically occurring on Fridays) when many prisoners are running out of tobacco, whereas the earlier study included samples taken at the weekend (after tobacco delivery). This earlier study reported that $PM_{2.5}$ concentrations were higher immediately after canteen delivery days [14]. It is also possible however that three months before going smoke-free, prisoners were already starting to reduce their tobacco consumption or had been on a smoking cessation course at the prison in light of the impending policy. Further validation of SHS levels recorded in this study pre-policy comes from two further air quality monitoring studies carried out in Scottish prisons prior to their smoke-free policy [13, 21] which produced similar pre-policy $PM_{2.5}$ concentrations.

Since 2005 the USA, New Zealand, Canada, and Australia have all implemented smoke-free policies in their correctional facilities. International air quality studies from New Zealand and the US have shown that comprehensive prison smoke-free polices are effective in substantially reducing SHS concentrations [20, 22, 26]. All of these studies used markers of SHS, respirable particulate matter (for example, particulate matter less than 2.5 µg/m³ ($PM_{2.5}$)) [20, 22] and airborne nicotine [26] to sample prison locations pre- and post- policy. The percentage reductions in $PM_{2.5}$ concentrations in our study were very similar to those recorded in these other countries [20, 22, 26].

For peer review only

Alongside reduced SHS concentrations, the potential health benefits of introducing a comprehensive smoke-free policy have been outlined in a study which examines the 10 years since the US implemented its smoking ban in prisons. This study found that prisons which implemented a smoke-free policy had a 9% reduction in smoking related deaths (particularly cardiovascular and pulmonary deaths), and that bans in place for longer than nine years were associated with a reduction in cancer deaths [27]. A study exploring natural deaths in male prisoners over 60 years of age in England and Wales reported diseases of the circulatory system (such as, coronary heart disease and cerebrovascular disease) and respiratory illnesses, all of which are substantially more common among smokers, as the most common cause of death [28]. With these findings in mind, the roll-out of a comprehensive smoke-free policy across all 121 prisons in England and Wales set out by HMPPS [15] has the potential to have the same positive health impact on the nearly 83,000 prisoners [29] currently held, and the 32,000 staff members employed [30], in the prison estate.

Findings from this study suggest prisoners were still smoking after the introduction of smoke-free, since $PM_{2.5}$ concentrations post- policy ranged from 0-3073 $\mu g/m^3$, consistent with continued smoking in some areas. Hammond and colleagues measured nicotine concentrations before and after prisons in California, US went smoke-free, and concluded that a smoking ban was effective in reducing SHS exposure but did not eliminate it [26]. An ethnographic case study conducted in ten prisons in the US after implementing a complete smoking ban described the lengths prisoners would go to in order to acquire, exchange and smoke tobacco, and how tobacco had now become a more lucrative commodity to sell due to big demand and higher profit margin than illicit drugs [31]. The study concluded that although prisoners smoked less post-policy, the emergent black-market created by banning tobacco had a negative impact on inmates. The emergence of a tobacco black market was also observed in New Zealand and the Northern Territories of Australia after their implementation of smoke-free [31-33].

Conclusion

Smoke-free policies in these prisons has successfully reduced prisoner smoking, and both prisoner and staff exposure to SHS. Further work to reduce still further the occurrence of prisoner smoking is clearly required, and to assess the impact of the smoke-free policy on prisoner health. However, our data provide strong evidence in support of the continued implementation of the smoke-free policy throughout the English and Welsh prison estate and in other penal systems internationally.

For peer review only

Security clearance

Researchers (LJ & CH) were security cleared to Enhanced Level 1, enabling them to visit establishments and work within NOMS. Researchers were given security talks from each establishments prior to data collection.

Authors' contributors

LJ is the guarantor and takes responsibility for the integrity of the work as a whole, from inception to publication; study conception and design, data collection, analysis of data, interpretation of data and drafting the research manuscript. RM, ER and JB contributed to the study conception and design. CH assisted with data collection and MOB supported data preparation in STATA and data analysis. RM and JB aided drafting the manuscript. All authors read and approved the final manuscript.

Acknowledgements

The lead author would like to thank all four prisons for agreeing to participate in this research study, alongside the staff members who escorted the researchers during data collection.

Funding

This work was supported by Medical Research Council (grant number MR/K023195/1) for the UK Centre for Tobacco and Alcohol Studies, which includes funding from the British Heart Foundation, Cancer Research UK, Economic and Social Research Council and the Department of Health under the auspices of the UK Clinical Research Collaboration. The funders had no role in the study design, data collection and analysis, decision to publish or preparation of the manuscript.

Data Sharing Statement

All available data can be obtained by contacting the corresponding author.

Competing Interests

None declared.

For peer review only

LEGEND

Figure 1. Box plots of $PM_{2.5}$ distributions in each of the four prisons pre- and post-smoke-free implementation.

Figure 2. Concentrations of $PM_{2.5}$ sampled on a main residential wing at HMP 3 pre- and post-smoke-free implementation.

For peer review only

REFERENCES

1. Bauld L. The impact of smokefree legislation in England: Evidence review. Available from: https://www.gov.uk/government/uploads/system/uploads/attachment_data/file/216319/dh_124959.pdf. 2011.

2. Department of Health. Smoke-free England - one year on. Available from: http://www.smokefreeengland.co.uk/files/dhs01_01-one-year-on-report-final.pdf London: UK. 2008.

3. Local Government Association. A breath of fresh air: Smokefree workplaces 10 years on. London: UK. June 2017

4. HM Government. Health Act 2006. Chapter 28, Smoking. Great Britain. 2006

5. Prison Service Instruction 09/2007. London: Smoke free legislation: prison service application. 2007.

6. Department of Health and HM Prison Service. Acquitted: Best practice guidance for developing smoking cessation services in prisons. London. 2003.

7. Lester C, Hamilton-Kirkwood L, and Jones N, Health indicators in a prison population: asking prisoners. Health Education Journal 2003;62:341-349.

8. Scottish Prison Service. Prisoner Survey 2015. Scottish Prison Service Scotland 2015.

9. Singleton, N., M. Farrell, and H. Meltzer. Substance misuse among prisoners in England and Wales. Available from: http://webarchive.nationalarchives.gov.uk/20160105160709/http://ons.gov.uk/ons/rel/psychiatric-morbidity/substance-misuse-among-prisoners/substance-misuse-among-prisoners/index.html. 1999.

For peer review only

10. Plugge EH, Foster CE, Yudkin PL, Douglas N, Cardiovascular disease risk factors and women prisoners in the UK: the impact of imprisonment. Health Promotion International. 2009; 24(4):334-343.

11. Prison Officers Association, Health and Safety Smoke Free. http://www.poauk.org.uk/index.php?circulars&newsdetail=20111114-185_health-safety-smoke-free-prison-estate. Prison Circular 2011.

12. Black -v- Secretary of State for Justice (Case No: CO/1258/2014): Hearing before the High Court Of Justice. 5 March 2015: Available from: https://www.judiciary.gov.uk/judgments/black-v-secretary-of-state-for-justice/.

13. Parsons Brinckerhoff. Report on Second-Hand Smoke in Prisons: Final Report. Available at - https://www.gov.uk/government/publications/air-quality-reports. 2015.

14. Jayes LR, Ratschen E, Murray RL, Dymond-White S, Britton J. Second-hand smoke in four English prisons: an air quality monitoring study. BMC Public Health. 2016;16(1):119.

15. Ministry of Justice. Ministry of Justice, Andrew Selous MP and National Offender Management Service. Letter from Prisons Minister Andrew Selous to Robert Neill MP, Chairman of the Justice Select Committee regarding smoking in prisons. September 2015.

16. The National Offender Management Service (NOMS). Prison Annual Performance Ratings 2015/16. Available from: https://www.gov.uk/government/uploads/system/uploads/attachment_data/file/541173/prison-annual-performance-ratings-2015-2016.pdf.

17. Hyland A, Travers MJ, and Dresler C. A 32-country comparison of tobacco smoke derived particle levels in indoor public places. Tobacco Control. Jun 2008;17(3):159-165.

18. Sureda X,Fernandez E, and Lopez MJ. Secondhand tobacco smoke exposure in open and semi-open settings: a systematic review. Environ Health Perspect. Jul 2013;121(7):766-73.

For peer review only

19. Semple S, Apsley A, Galea KS, MacCalman L, Friel B, Snelgrive V. Secondhand smoke in cars: assessing children's potential exposure during typical journey conditions. Tobacco Control. Nov 2012;(6):578-83.

20. Proescholdbell SK, Foley KL, and Johnson J. Indoor air quality in prisons before and after implementation of a smoking ban law. Tobacco Control. Apr 2008;(2):123-7.

21. Semple S, Sweeting H, Demou E, Logan G, O'Donnell R, Hunt K. Characterising the Exposure of Prison Staff to Second-Hand Tobacco Smoke. Annals of Work Exposures and Health. 2017;61(7):809-821.

22. Thornley S, Dirks KN, Edwards R. Indoor air pollution levels were halved as a result of a national tobacco ban in a New Zealand prison. Nicotine Tob Res. Feb 2013;15(2):343-7.

23. Jiang RT, Acevedo-Bolton V, and Cheng KC. Determination of response of real-time SidePak AM510 monitor to secondhand smoke, other common indoor aerosols, and outdoor aerosol. J Environ Monit. Jun 2011;13(6):1695-702.

24. Semple S, Tongeren M, and Galea KS. UK smoke-free legislation: changes in PM2.5 concentrations in bars in Scotland, England, and Wales. Ann Occup Hyg. Apr 2010;54(3):272-80.

25. WHO air quality guidelines. Global Update. Geneva, Switzerland. 2005.

26. Hammond SK, and Emmons KM. Inmate exposure to secondhand smoke in correctional facilities and the impact of smoking restrictions. J Expo Anal Environ Epidemiol. 2004;15(3):205-211.

27. Binswanger I, Carson E, Krueger P, Mueller S, Steiner J, Sabol W. Prison tobacco control policies and deaths from smoking in United States prisons: population based retrospective analysis. BMJ. 2014;349:g4542.

28. Fazel S, and Benning R. Natural deaths in male prisoners: a 20-year mortality study. European Journal of Public Health. 2006;16(4):441-444.

29. Ministry of Justice. National Offender Management Service (NOMS), Prison population figures: 2018. Available from: https://www.gov.uk/government/statistics/prison-population-figures-2018. 2018.

For peer review only

30. Ministry of Justice. Her Majesty's Prison and Probation Service workforce quarterly: June 2017, Workforce Statistics Bulletin, 2017: Available from: https://www.gov.uk/government/statistics/her-majestys-prison-and-probation-service-workforce-quarterly-june-2017. 2017.

31. Lankenau SE. Smoke 'em if you got 'em: Cigarette black markets in U.S. prisons and jails. The Prison journal. 2001;81(2):142-161.

32. Collinson L, Wilson N, Edwards R, Thomson G, Thornley S. New Zealand's smokefree prison policy appears to be working well: one year on. The New Zealand Medical Journal. 2012; 125(1357):164-168.

33. Hefler MR, Hopkins R, and Thomas D. Successes and unintended consequences of the Northern Territory's smoke-free prisons policy: results from a process evaluation. Public Health Research & Practice. Apr 2016;26(2)e26216219.

For peer review only

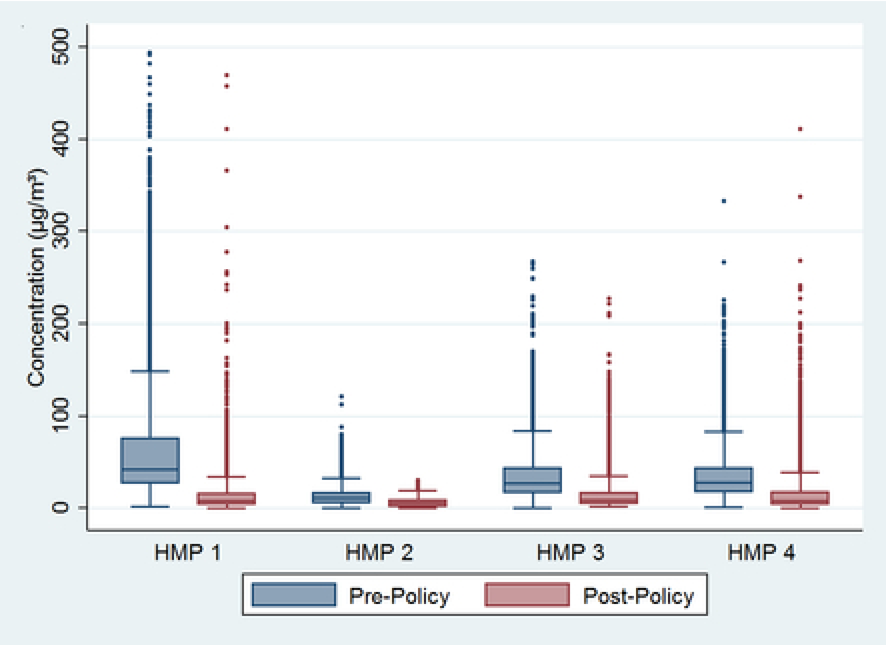

37x27mm (300 x 300 DPI)

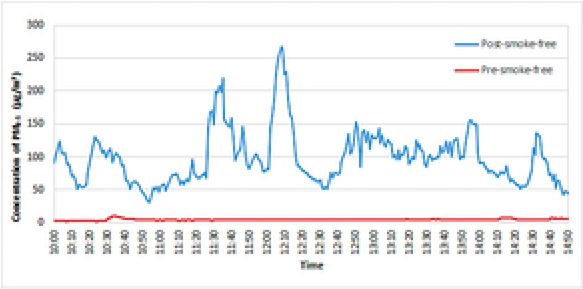

24x12mm (300 x 300 DPI)

STROBE checklist

Section	Page number	Explanatory Notes
**Title**	Pg. 1	Smoke-free prisons in England: Indoor air quality before and after implementation of a comprehensive smoke-free policy
**Abstract**	Pg. 2/3	Please refer to abstract section of the manuscript
**Introduction**		
Background	Pg.3/4	Refer to the 'Introduction' section of the manuscript for a brief background of the topic
Objectives	Pg.3/4	By measuring concentrations of airborne particulate matter <2.5 microns in diameter ($PM_{2.5}$) as a proxy for second-hand smoke (SHS), this study intends to determine to what extent a new smoke-free policy reduces concentrations of SHS in the first four prisons to go smoke-free in England.
**Methods**		
Study design	Pg. 1/2/5	Air quality monitoring study. In the title of the study and section 'design' in Abstract
Setting	Pg. 5/9	Great Britain, four prisons in the South-West of England. In 'Study prison' and Table 1 provides further information on each prison and outlines dates of data collection.
Participants	N/A	Our interest is in particulate matter pre- to post- smoke-free policy.
Variables	Pg. 6	Section 'particulate pollution' in Methods
Data source	Pg. 5/6	Section 'study prisons and particulate pollution' in Methods
Bias	Pg. 10/17	Section 'data analyses' in Methods and in Discussion section. Air quality measurements were not carried out blind and seasonal differences may have also impacted on the sample concentrations (winter v. summer). Where possible, placed monitors away from other sources of $PM_{2.5}$.
Study Size	Pg. 2/5	In 'abstract', 'introduction' and 'study prisons' sections. The prison service decision to pilot four smoke-free sites, all were sampled.
Quantitative variables	Pg. 10	Section 'data analysis' in Methods. Pre and post particulate matter were matched for prison, wing location and monitor placement, day of the week and time.
Statistical method	Pg. 10	Section 'data analysis' in Methods
**Results**		
Participants	N/A	Our interest is concentrations of airborne particulate matter <2.5 microns in diameter ($PM_{2.5}$) measured as a proxy for second-hand smoke (SHS).
Descriptive data	Pg.	Section 'results'- 74 paired sets of data collected over 29 wing locations in the four prisons sampled. Sampling $PM_{2.5}$ for an average of 5 hours and 8 minutes.
Outcome data	Pg. 11	Section 'combined data for all four prisons, comparing PM2.5 concentrations collected pre-and post-
Main Results	Pg. 11	implementation' Section 'combined data for all four prisons, comparing PM2.5 concentrations collected pre-and post-implementation'. Mean $PM_{2.5}$ concentrations on wing landing locations before the introduction of smoke-free policy were 39 µg/m³, and 13 µg/m³ after introduction, representing a 66% reduction in mean $PM_{2.5}$ concentrations (mean difference 26 µg/m³, 95% Confidence Intervals 25

		μg/m³ to 26 μg/m³); and a 69% reduction in median $PM_{2.5}$ concentrations (from 26 μg/m³ to 8 μg/m³).
Other analysis	Pg. 11/14	Results section 'combined data for all four prisons, comparing PM2.5 concentrations collected pre-and post-implementation' provide comparisons to WHO guidance levels and 'individual data from all four prisons, comparing $PM_{2.5}$ concentrations collected pre-and post-implementation' provides breakdown of results per prison.
**Discussion**		
Key Results	Pg. 16	First paragraph of the Discussion section.
Limitations	Pg.16/17/18	Paragraphs 2-4 in Discussion section.
Interpretation	Pg. 18	Paragraph 4 in Discussion - our data does give a very good estimation, in view of the large amount of matched data collected pre- and post- policy (over 15 days pre- and post-policy), of SHS pollution during times when non-smokers would be exposed during waking hours.
Generalisability	Pg. 20	Paragraph 6 and 'conclusion' in Discussion
**Other Information**		
Funding	Pg. 21	This study was funded by a UK Centre for Tobacco and Alcohol Studies (UKCTAS) PhD studentship.

For peer review only
